# Supplementary material for: Minutes-timescale 3D isotropic imaging of entire organs at subcellular resolution by content-aware compressed-sensing light-sheet microscopy
Source: Nat Commun. 2021 Jan 4;12:107. doi: 10.1038/s41467-020-20329-3 (PMC7782498; doi:10.1038/s41467-020-20329-3)
Supplement: Supplementary file 1 — Supplementary Information [file 41467_2020_20329_MOESM1_ESM.pdf]

Supplementary Information

Supplementary Figure 1 | Dual-side, dual-mode light-sheet setup

Supplementary Figure 2 | The mechanical design for sample holding

Supplementary Figure 3 | Comparison of different plane illumination modes

Supplementary Figure 4 | Necessity for line synchronization under different magnification/NA setups

Supplementary Figure 5 | Timing diagram of for line synchronization and unsynchronized dithering modes

Supplementary Figure 6 | CACS computation for line-like neurons with different signal density at lateral planes

Supplementary Figure 7 | CACS computation for line-like neurons with different signal density at axial planes

Supplementary Figure 8 | CACS computation for point-like cell nuclei with different signal density at lateral planes

Supplementary Figure 9 | CACS computation for point-like cell nuclei with different signal density at axial planes

Supplementary Figure 10 | CACS computation for resolving finer neuronal sub-structures

Supplementary Figure 11 | CACS computation for images obtained by confocal and TPE microscopes

Supplementary Figure 12 | Signal-to-noise-ratio (SNR) comparison of different modes

Supplementary Figure 13 | Photobleaching rate comparison of different imaging modes

Supplementary Figure 14 | Scalable isotropic imaging of neurons in mouse brain

Supplementary Figure 15 | Tracing dense neurons in the cortex area of mouse brain (Thy1-GFP-M, 8 weeks)

Supplementary Figure 16 | 3D registration, region segmentation and neuron tracing of whole mouse brain (Thy1-GFP-M, 8 weeks)

Supplementary Figure 17 | Accuracy of compressed sensing in PI-labelled brain

Supplementary Figure 18 | Segmentation and cell counting for PI-labelled half brain imaged by 2× CACS Bessel sheet

Supplementary Figure 19 | The limit of CACS recovery

Supplementary Table 1 | Full part list of our dual-side, dual-mode light-sheet microscope

Supplementary Note 1 | Imaging speed, photobleaching rate and SNR

Supplementary Note 2 | Image stitching and dual-view image fusion

Supplementary Note 3 | Content aware regularization in CS

Supplementary Note 4 | Synthetic PSF and measurement matrix *A* in CACS

Supplementary Note 5 | An interior-point method to iteratively solve equations

Supplementary Note Figure 1 | Content-aware calculation of regularization factor

Supplementary Note Figure 2 | Generate a synthetic PSF and measurement matrix *A*

Supplementary Note Table 1 | Comparison of different imaging modes

Supplementary Note Table 2 | Whole brain imaging with different magnification

Supplementary Movie 1 | Confocally-scanned Bessel light-sheet microscopy

Supplementary Movie 2 | Content aware compressed sensing (CACS) procedure

Supplementary Movie 3 | Super-resolution of line-like neuron fibers by CACS

Supplementary Movie 4 | 3D imaging of whole mouse brain (neuron tagged) by CACS Bessel sheet microscopy

Supplementary Movie 5 | Whole-brain 3D visualization and segmentation

Supplementary Movie 6 | Accuracy verification of tracing neuronal projections in the entire brain

Supplementary Movie 7 | Tracing long-distance neuronal projections across the entire brain

Supplementary Movie 8 | Super-resolution of point-like cell nuclei by CACS

Supplementary Movie 9 | 3D imaging of half mouse brain (nuclei stained) by CACS Bessel sheet microscopy

Supplementary Movie 10 | Region-specific cell counting in half mouse brain

Supplementary Movie 11 | Dual-color 3D imaging of mouse gastrocnemius and tibialis muscles by CACS Bessel sheet microscopy

Supplementary Movie 12 | Neuron tracing and MEP counting in gastrocnemius muscle

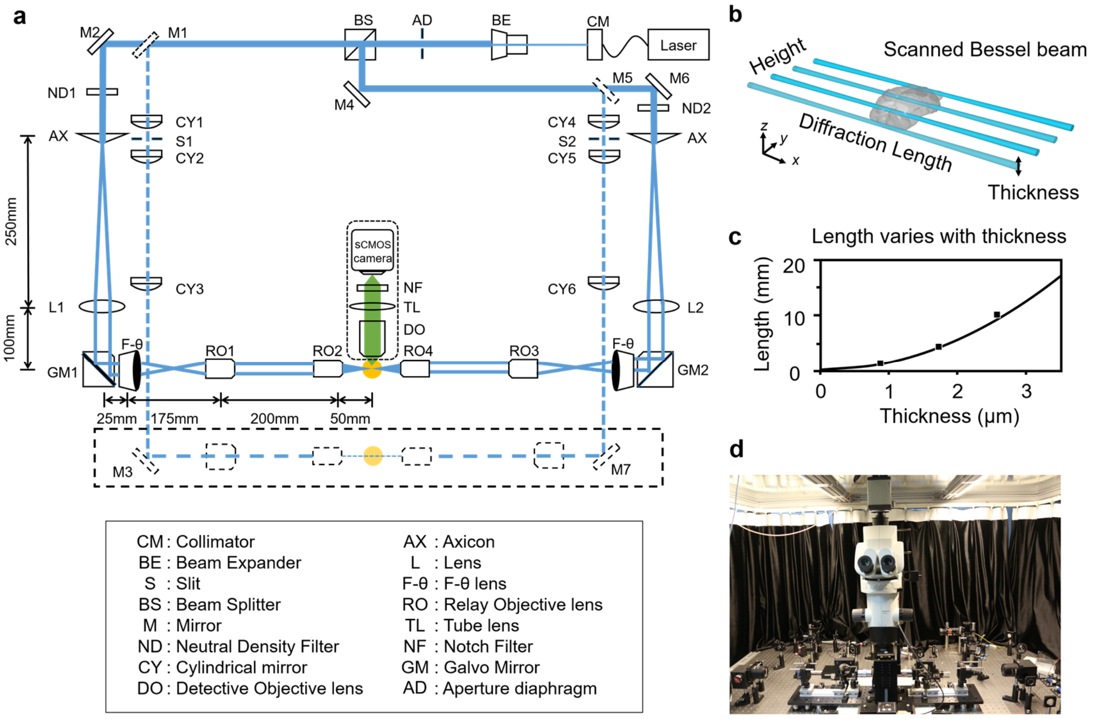

Supplementary Fig. 1 | Dual-side, dual-mode light-sheet setup. a, A multi-wavelength laser was collimated and expanded to generate a Gaussian beam with 10 mm diameter. For each side, an axicon (AX) was used to generate a Bessel beam, which was further scanned into a plane by a galvo scanner (GM) and projected onto the sample using two groups of relay lenses (L1, L2, F-theta lenses, RO1-4). Dual Bessel sheets were precisely aligned by finely tuning the *y*-mirror of GM2. Finally, a Bessel plane illumination with widely tunable geometry (1-5 μm thickness, 1-20 mm FOV width, 1-10 mm height) was formed for rapid and high-axial-resolution imaging of large specimen. In our setup, a dual-side Gaussian light sheet (shown in the dashed box, switched by M1, 3, 5, 7) was also reserved for quick sample screening, as well as comparison with Bessel sheet. b, c, Geometry of scanning Bessel sheet and the correlation between its thickness (axial resolution) and diffraction length (width of FOV). d, Photograph of the microscope built on an optical bench.

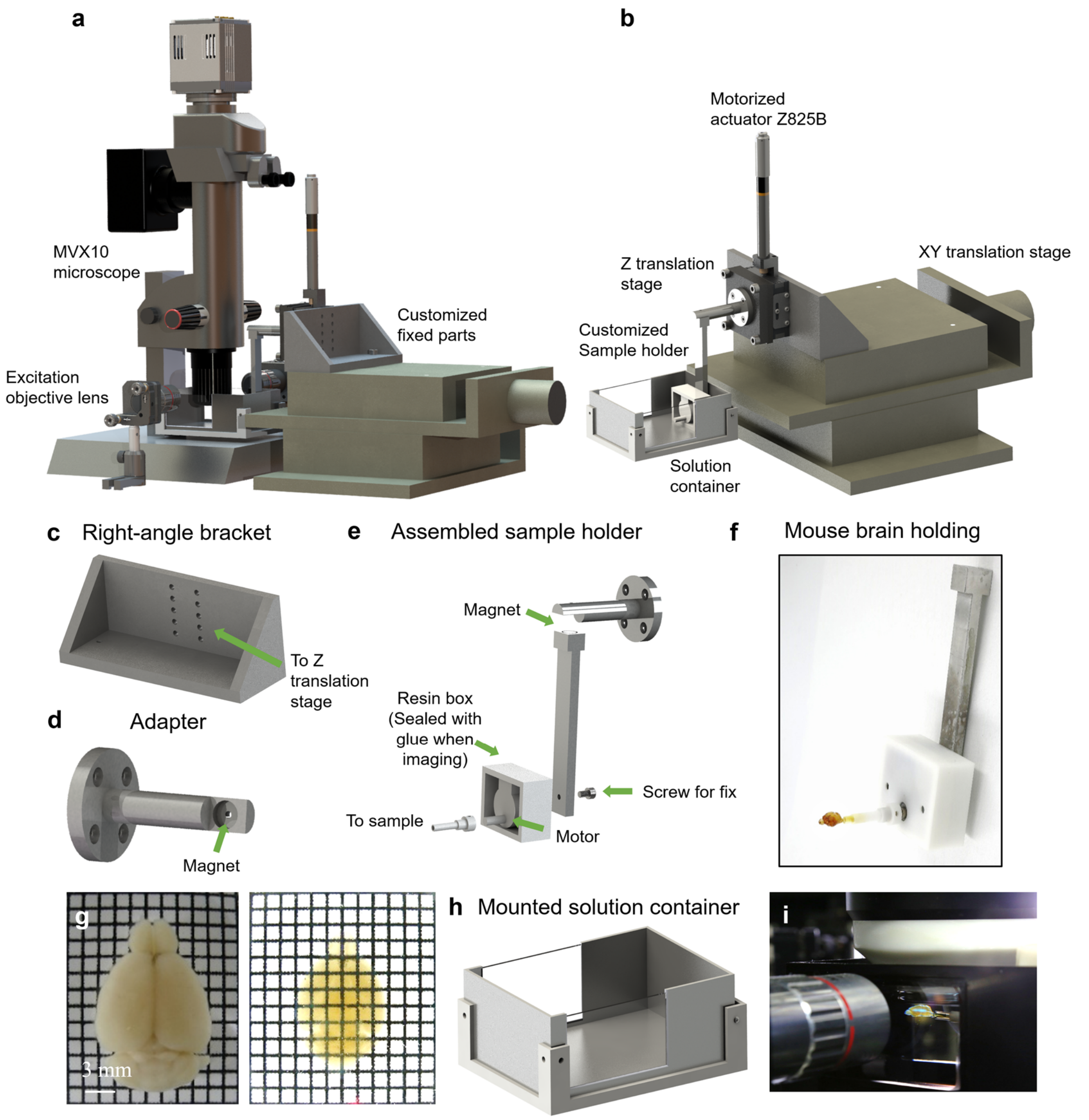

Supplementary Fig. 2 | The mechanical design for sample holding. a, 3D layout of assembled sample mounting, translation and fluorescence detection parts. b, Detailed view of the sample mounting and 3D translation. A right-angle bracket (shown in c) was used to integrate the *z* translation stage with the *xy* stage, and a customized magnetic connector (shown in d) was designed to connect the 3D translation stage with the sample holder (shown in e). e, Detailed design of the sample holder. The cleared sample was mounted to the shaft of a water-proof motor (2, sealed in a resin box) through a FEP tube connector (1). The motor mounted with sample was then fixed with a rod-like magnet holder (3) using set screw. Such a sample holder containing brain sample and 1,2,3 parts can be readily attached to the magnetic connector (4, also shown in d) by using magnetic force. This design permits: 1. 3D translation and rotation of the sample in the solution. 2. convenient sample loading/unloading through the magnetic force. f, Photograph of the sample holder corresponding to the design layout shown in e. g, Photographs of an excised whole mouse brain before and after tissue clearing. h, Design of the solution container and its mounting part. i, Photograph showing that the scanned Bessel sheet is illuminating a transverse plane of the cleared mouse brain, and the detection objective is simultaneously collecting the fluorescence signals excited at the illuminated plane.

Supplementary Table 1 | Full part list of our dual-side, dual-mode light-sheet microscope

Item #	Part name	Product #	Quantity	Vendor
1	Multi-wavelengths Laser System AT 637&532&488nm	RGB-637/532/488nm-60mW	1	Changchun New Industries
2	3X Achromatic Galilean Beam Expander	GBE03-A	1	Thorlabs
3	Motorized Filter Flip Mount	MFF101/M	1	Thorlabs
4	Lens Mount	LMR1/M	1	Thorlabs
5	Kinematic, SM1-Threaded, 30 mm-Cage-Compatible Mount with Slip Plate for Ø1" Optic, Metric	KC1-S/M	2	Thorlabs
6	Locking Kinematic Mirror Mount for Ø2" Optics	KC2/M	2	Thorlabs
7	2.0°, Ø1" (Ø25.4 mm) Axicon	AX252-A	2	Thorlabs
8	f=100 mm, Ø1" Achromatic Doublet	AC254-100-A-ML	2	Thorlabs
9	f=75 mm, Ø1" Achromatic Doublet	AC254-075-A-ML	2	Thorlabs
10	f=80 mm, Ø2" Achromatic Doublet	AC508-080-A	2	Thorlabs
11	30 mm Cage Adapter	GCM012/M	2	Thorlabs
12	Scan Lens, 400 to 750 nm, EFL=70 mm	CLS-SL	2	Thorlabs
13	Adapter	SM2A20	2	Thorlabs
14	Tube Lens, f = 200 mm	ITL200	2	Thorlabs
15	SM1-Threaded Kinematic Mount	KM100T	2	Thorlabs
16	SM1-Threaded 30 mm Cage Plate	CP33/M	2	Thorlabs
17	30 mm Cage Plate	CP35/M	2	Thorlabs
18	SM1-Threaded 30 mm Cage Plate	CP33T/M	1	Thorlabs
19	60 mm Cage Plate	LCP01T/M	2	Thorlabs
20	30 mm Cage Plate	CP36	2	Thorlabs
21	60 mm Cage Plate	LCP01/M	2	Thorlabs
22	Cage Assembly Rod	ER3	4	Thorlabs
23	Cage Assembly Rod	ER2	4	Thorlabs
24	Ø25.0 mm Pedestal Pillar Post	RS6P/M	2	Thorlabs
25	Ø25.0 mm Pillar Post	RS12/M	12	Thorlabs
26	Clamping Fork	CF175	2	Thorlabs
27	Ø12.7 mm Optical Post, L = 20 mm	TR20/M	4	Thorlabs
28	Ø12.7 mm Optical Post, L = 30 mm	TR30/M	3	Thorlabs
29	Ø12.7 mm Optical Post, L = 40 mm	TR40/M	2	Thorlabs
30	Ø12.7 mm Optical Post, L = 50 mm	TR50/M	2	Thorlabs
31	Ø12.7 mm Optical Post, L = 75 mm	TR75/M	1	Thorlabs
32	Ø12.7 mm Universal Post Holder, L = 30 mm	UPH30/M	2	Thorlabs
33	Ø12.7 mm Universal Post Holder, L = 40 mm	UPH40/M	3	Thorlabs
34	Ø12.7 mm Universal Post Holder, L = 50 mm	UPH50/M	5	Thorlabs
35	Ø12.7 mm Universal Post Holder, L = 75 mm	UPH75/M	2	Thorlabs
36	Ø12.7 mm Post Holder, L=20 mm	PH20/M	6	Thorlabs
37	Swivel Base Adapter	UPHA	2	Thorlabs
38	25 mm Motorized Actuator	Z825B	1	Thorlabs

39	K-Cube Brushed DC Servo Motor Controller (Power Supply Not Included)	KDC101	1	Thorlabs
40	Ø1" Protected Silver Mirror	PF10-03-P01	10	Thorlabs
41	Ø2" Protected Silver Mirror	PF20-03-P01	4	Thorlabs
42	66 mm Single Dovetail Rail, L = 500 mm	XT66SD-500	1	Thorlabs
43	Double Dovetail Rotation Clamp	XT66RD	6	Thorlabs
44	Mounting Platform	XT66D2-50	6	Thorlabs
45	Complete Periscope Assembly, Metric (Mirrors Not Included)	RS99/M	2	Thorlabs
46	90° Flip Mount for Ø1" Filters and Optics, M4 Tap	TRF90/M	2	Thorlabs
47	Kinematic Mount for up to 1.3" (33 mm) Tall Rectangular Optics, Right Handed	KM100C	6	Thorlabs
48	f = 40.00 mm, H = 20.00 mm, L = 22.0 mm, N-BK7 Plano-Convex Cylindrical Lens, Uncoated	LJ1125L1	2	Thorlabs
49	f = 100.00 mm, H = 30.00 mm, L = 32.0 mm, N-BK7 Plano-Convex Cylindrical Lens, Uncoated	LJ1567L1	2	Thorlabs
50	f = 300.12 mm, H = 30.00 mm, L = 60.0 mm, N-BK7 Plano-Convex Cylindrical Lens, Uncoated	LJ1558L2	2	Thorlabs
51	Long working distance objective lens	M Plan Apo 2X/0.055	2	Mitutoyo
52	Long working distance objective lens	M Plan Apo 5X/0.14	2	Mitutoyo
53	Long working distance objective lens	M Plan Apo 10X/0.28	2	Mitutoyo
54	Macro Zoom Fluorescence Microscope	MVX10	1	Olympus
55	sCMOS camera	ORCA-Flash4.0 V2	1	Hamamatsu
56	xy axis translation stage	SST59D3306	1	Huatian
57	Aluminum Breadboard, 100 mm x 150 mm x 12.7 mm	MB1015/M	2	Thorlabs
58	Aluminum Breadboard, 150 mm x 600 mm x 12.7 mm	MB1560/M	1	Thorlabs
59	Ø25.0 mm Pedestal Pillar Post, L = 12.5 mm	RS05P/M	12	Thorlabs
60	13 mm Translation Stage	MT1/M	2	Thorlabs
61	7" x 4" Lab Jack	L490/M	1	Thorlabs
62	30 mm Cage System Adjustable Slit	VA100C	2	Thorlabs
63	Right-angle bracket (Fig. S2c)	Customized	1	N/A
64	Adapter (Fig. S2d)	Customized	1	N/A
65	Rod-like magnet holder	Customized	1	N/A
66	Resin box	Customized	1	N/A
67	FEP tube connector	Customized	1	N/A
68	5V DC Motor	24BYJ48	1	YOFON

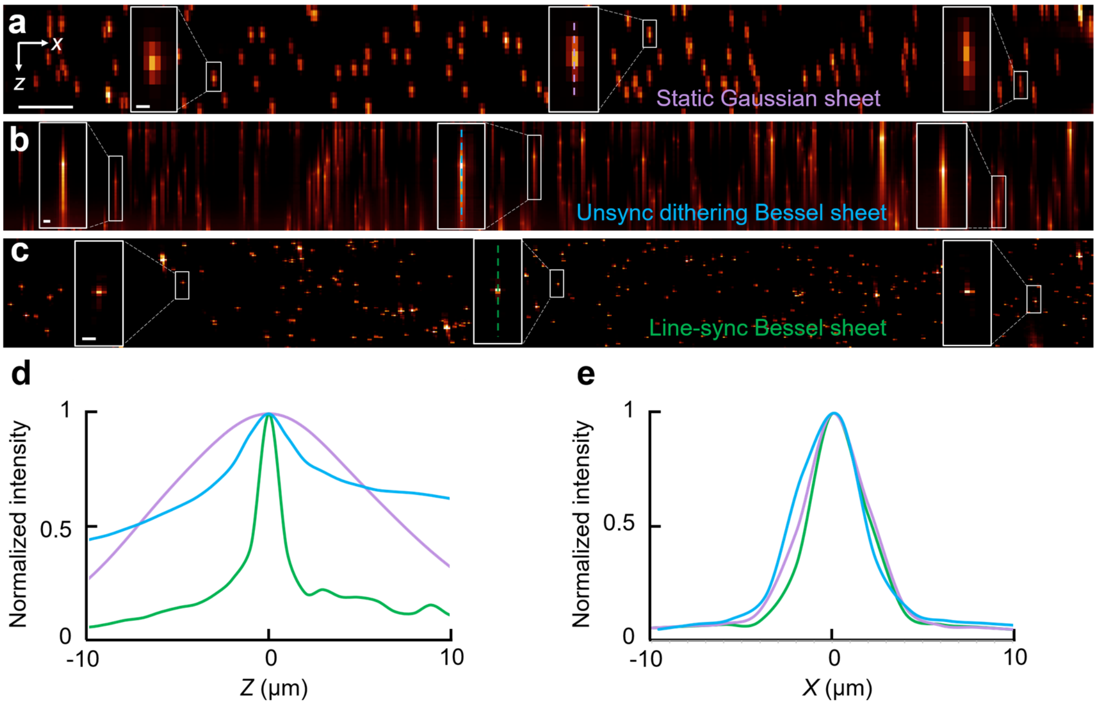

Supplementary Fig. 3 | Comparison of different plane illumination modes. We used electronic confocal slit to eliminate side lobe excitation from Bessel beam and thereby increase axial resolution. When compared to a 15 μm-thick regular Gaussian sheet under 3.2×/0.28 detection, unsynchronized (high-speed dithering) Bessel sheet caused substantively elongated PSF due to the accumulated axial excitation by side lobes, which cannot be eliminated by the large depth-of-field detection objective. In contrast, synchronizing scanned Bessel sheet with electronic slit significantly reduced this side effect, yielding isotropic PSF with axial extent much shorter than that of Gaussian sheet. a-c, PSFs measured by Gaussian sheet, unsynchronized Bessel sheet, and synchronized Bessel sheet modes, as shown in a-c, respectively. d, e, Axial and lateral line profiles of the beads resolved by three methods. It should be noted that since the *z*-scan step size was set to 0.5 μm (oversampling) for these PSF measurements, the axial FWHM of PSF by synchronized Bessel sheet (~1.6 μm) here corresponds to the native central-lobe thickness of Bessel sheet, which are thereby smaller than the axial FWHMs of neuron fibers (~4.5 μm in main text) measured using a 2-μm step size and even the lateral FWHM of PSF obtained by a 2-μm-pixel sampling. All plots were normalized in the same way. Scale bar, 50 μm (inset, 5 μm).

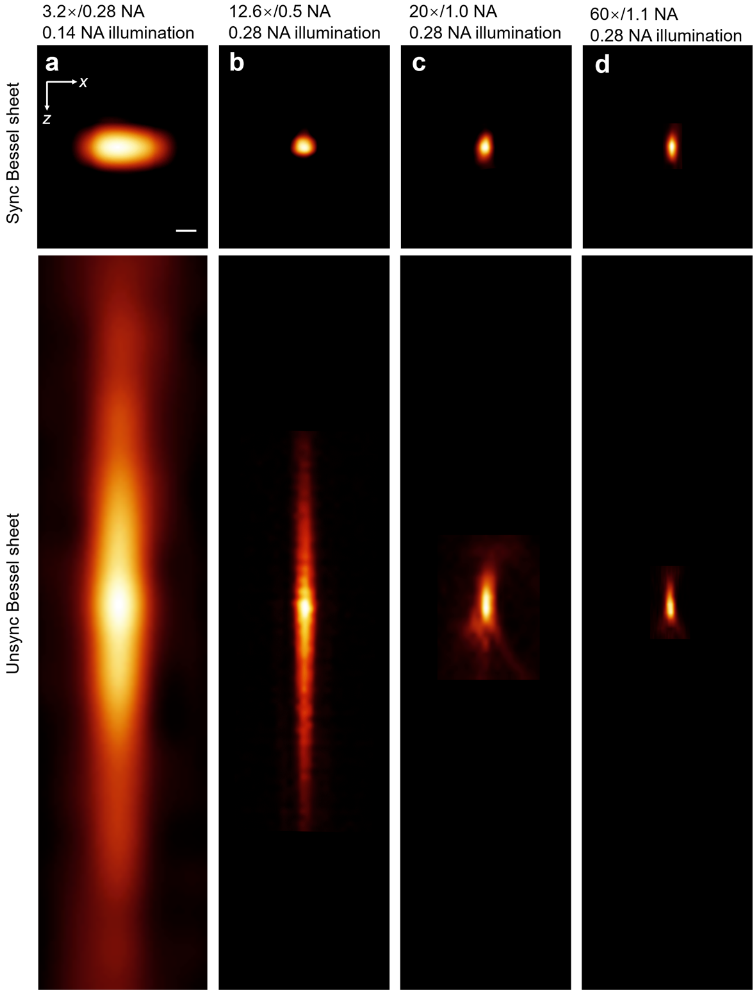

Supplementary Fig. 4 | Necessity for line synchronization under different magnification/NA setups. It should be noted that the axial fluorescence contamination from side-lobe excitation is especially severe under low (3.2×)-to-middle (12.6×) magnification, which is our case, due to the extended depth-of-focus. We have validated this through imaging the fluorescence beads (~0.2 μm) under 3.2×/0.28 and 12.6×/0.5 detection using both synchronized and unsynchronized Bessel sheet modes, and comparing their axial performance with those by 20×/1.0 and 60×/1.2 detections. a-d, Axial performance of the PSFs (*x*-*z* planes) under 3.2×/0.28, 12.6×/0.5, 20×/1.0 and 60×/1.2 detection setups. The top and bottom rows show the synchronized and unsynchronized results, respectively. Scale bar, 1 μm. While all four setups showed deteriorated axial excitation by side-lobe excitation when line synchronization was not applied, the 3.2×/0.28 and 12.6×/0.5 setups were obviously more vulnerable to this side effect, owing to their relatively large depth-of-focus that received excessive axial signals excited by the side lobes. Therefore, the line synchronization is particularly necessary for our implementation.

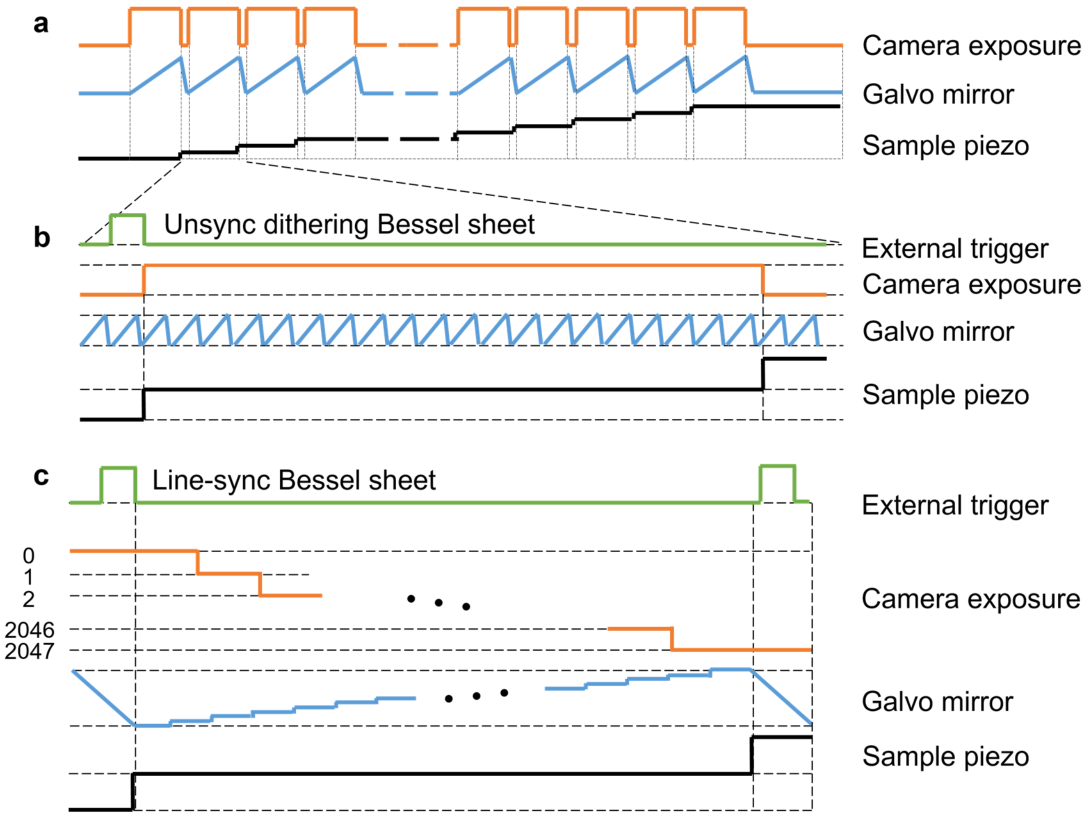

Supplementary Fig. 5 | Timing diagram of for line synchronization and unsynchronized dithering modes. a, Control signals for sequential multi-plane imaging. b, Control signals for unsynchronized Bessel sheet mode (high-speed dithering) at each plane. In this mode, the camera sensor stayed at global shutter while the beam was scanned back and forth at the sample plane with period much shorter than the exposure time. c, Control signals for synchronized Bessel sheet mode. In this mode, the camera sensor generated a rolling exposure line synchronized with the scanned central maximum of Bessel beam. When the beam was scanned from the top to the bottom of sample plane, the narrow active pixel line was simultaneously triggered for rolling with the same velocity and direction, so that the fluorescence signals excited by the central peak of Bessel beam could be always detected while those from the side lobes were always blocked.

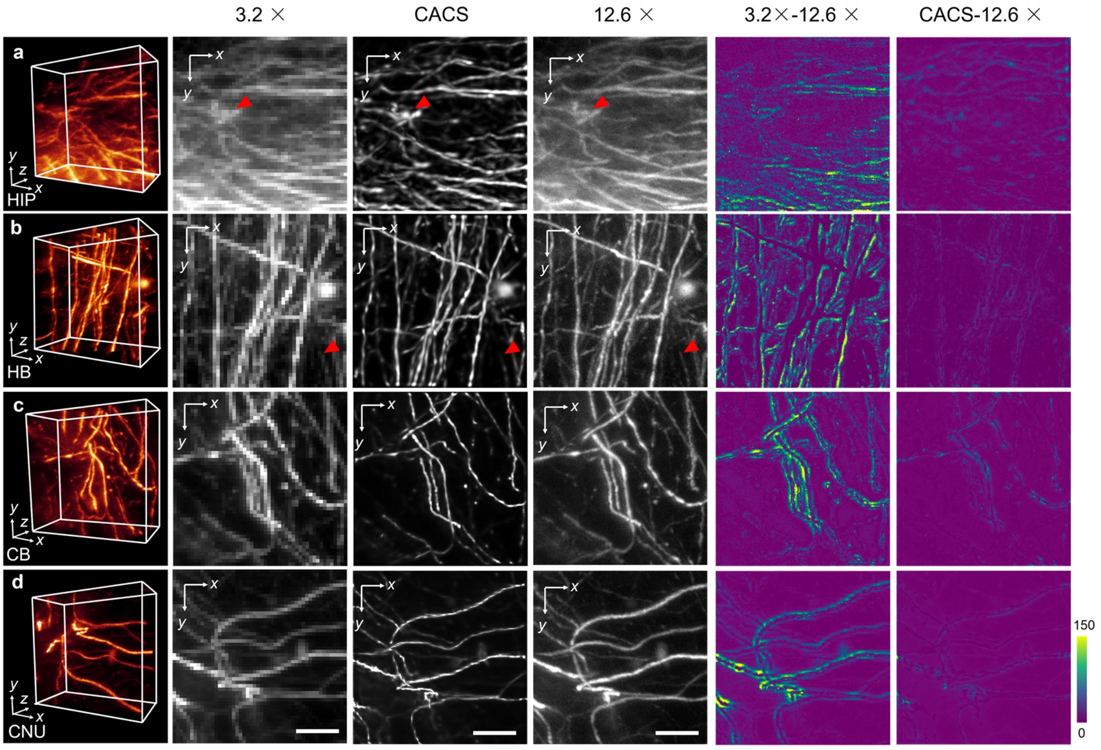

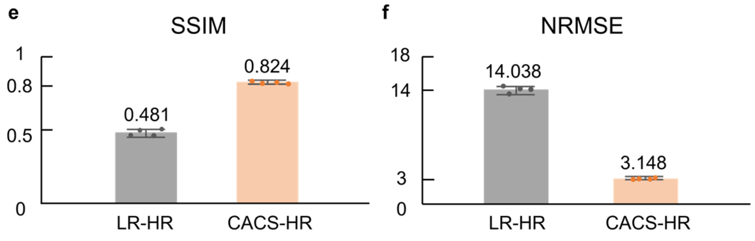

g

Region	α	β
HIP	1.1067	0.3984
HB	1.1641	0.4085
CB	1.3521	0.4217
CNU	1.3808	0.4318

Supplementary Fig. 6 | CACS computation for line-like neurons with different signal density at lateral planes. Four ROIs of whole mouse brain (Thy1-GFP-M) containing neuronal fibers with different signal densities were imaged using 3.2× CACS Bessel sheet. 12.6× Bessel sheet results were regarded as ground truth to validate the accuracy of CACS recovery. a-d, Comparisons between 3.2×, 3.2× CACS and 12.6× results of the four ROIs in HB, CNU, HIP and CB brain regions, respectively. The red arrows in the *x-y* planes indicated minor inaccurately-resolved signals by CACS, which were more likely to appear in dense signal regions. The error maps of 3.2× and 3.2× CACS shown at the right two columns also confirmed the high recovery accuracy of CACS. e-f, Using HR 12.6× results as reference, notably higher structural similarity (SSIM) and lower normalized root mean squared error (NRMSE) were obtained from 3.2× CACS results, as compared to LR 3.2× results (with 4× bicubic interpolation). Both SSIM and NRMSE were calculated based on the whole 3D volume. Data are presented as mean values +/- SD (n=4 biologically independent samples). The perceptual assessments together with quantitative analyses have validated the high recovery fidelity of CACS for line-like signals when providing resolution improvement. g, Different content-aware parameters applied for CS computation of the four regions. Each experiment was repeated 4 times independently with similar results. Scale bar, 20 µm.

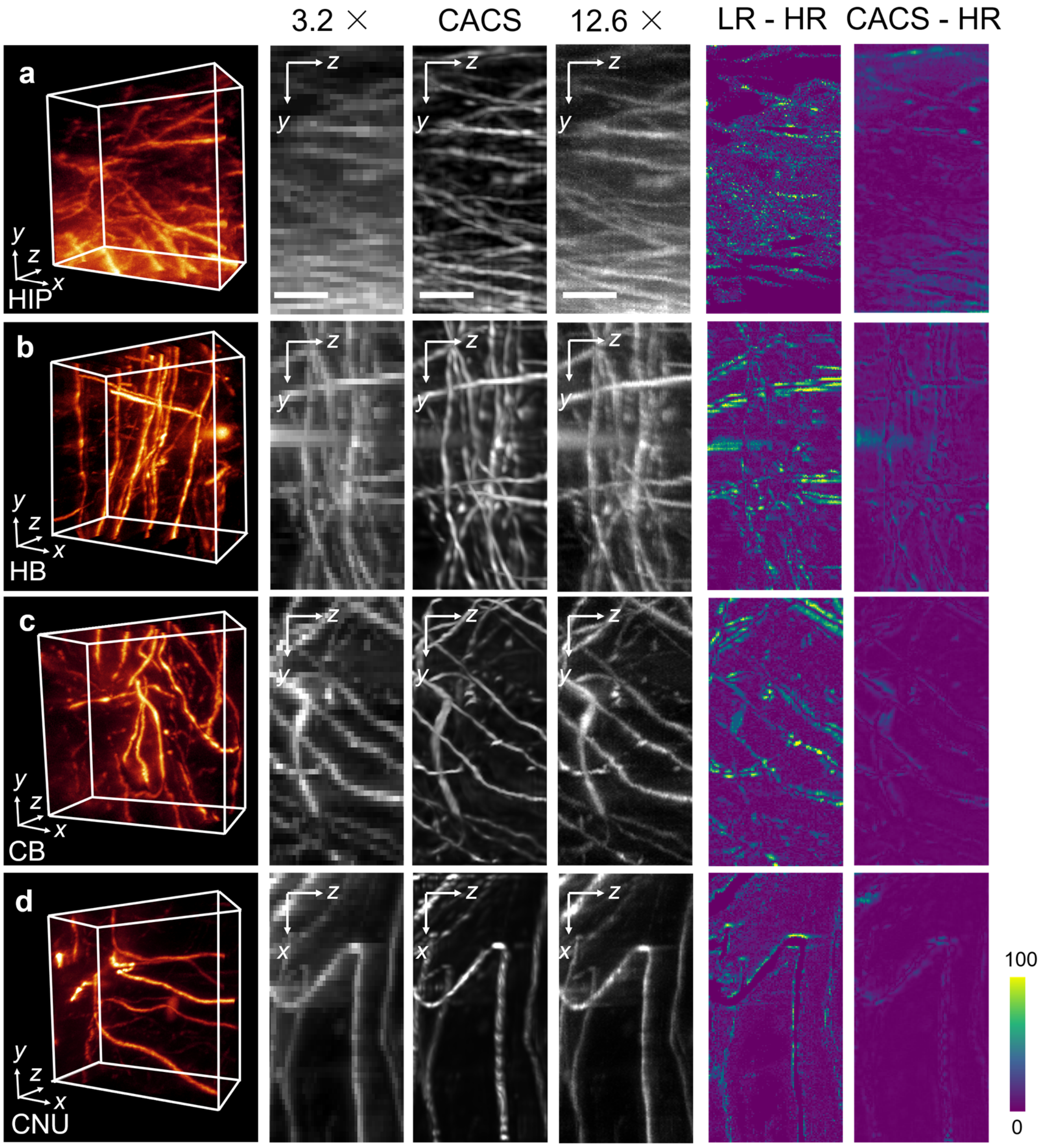

Supplementary Fig. 7 | CACS computation for line-like neurons with different signal density at axial planes. *x-z* planes of the same four volumes shown in Fig. S6 were shown to compare the difference at axial planes. a-d, Comparisons between 3.2×, 3.2× CACS and 12.6× results of the four volumes in HB, CNU, HIP and CB brain regions, respectively. The axial planes (*x-z* planes) were visually inspected in the second to fourth columns. The error maps of 3.2× and 3.2× CACS results shown at fifth and sixth columns further validated the high fidelity of axial planes in CACS results. Scale bar, 20 µm.

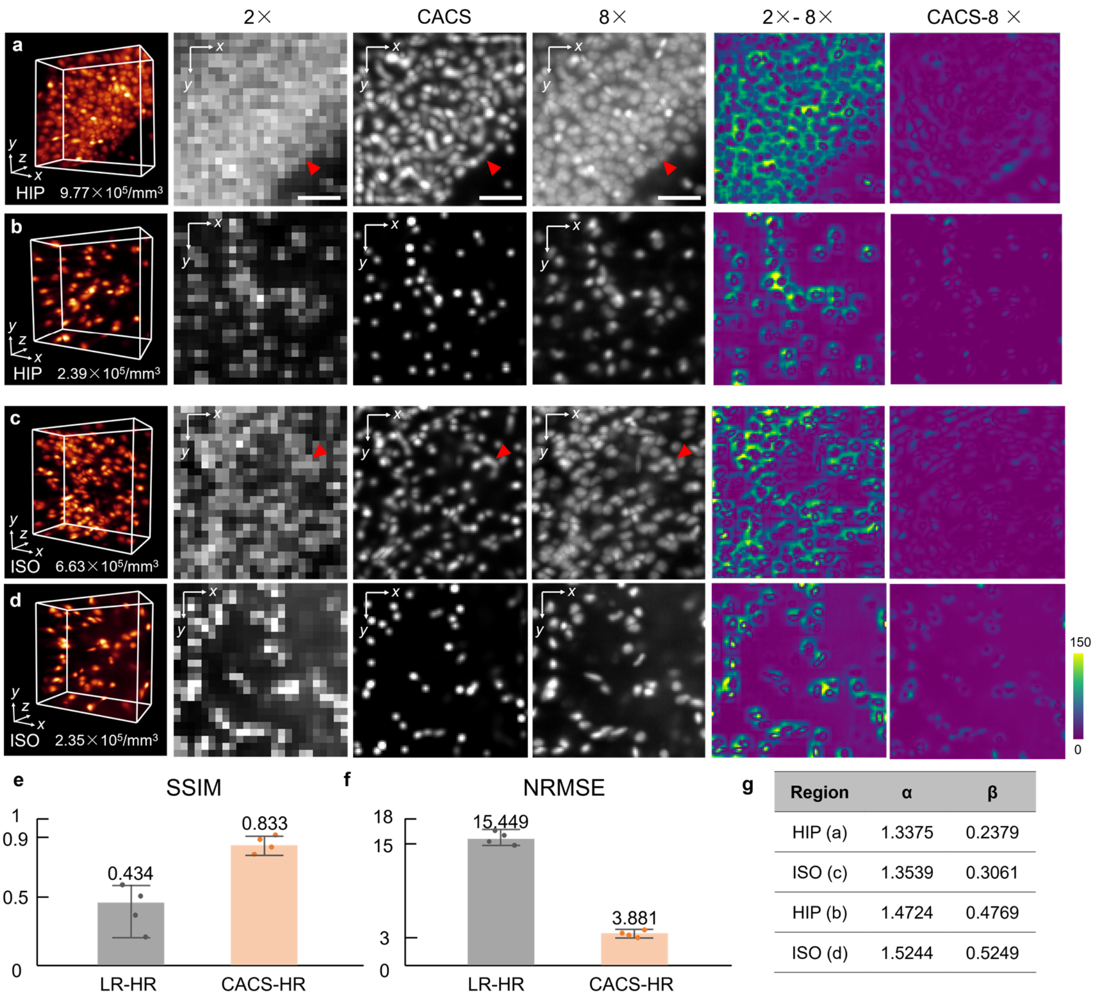

Region	α	β
HIP (a)	1.3375	0.2379
ISO (c)	1.3539	0.3061
HIP (b)	1.4724	0.4769
ISO (d)	1.5244	0.5249

Supplementary Fig. 8 | CACS computation for point-like cell nuclei with different signal density at lateral planes. a-d, Comparisons between 2×, 2× CACS and 8× results of the four volumes in PI-labelled hippocampus and isocortex, respectively. These ROIs contained cell nuclei with different density. The red arrows in the *x-y* planes of a and c showed minor inaccurately-resolved nuclei signals by CACS, which were also indicated in the error maps at the right columns. e-f, SSIM and NRMSE values of LR 2× results (with 4× bicubic interpolation) and 2× CACS results, calculated by using HR 8× results as reference. Data are presented as mean values +/- SD (n=4 biologically independent samples). The perceptual assessments together with quantitative analyses have validated the high recovery fidelity of CACS for point-like signals as well. Each experiment was repeated 4 times independently with similar results. Scale bar, 20 µm.

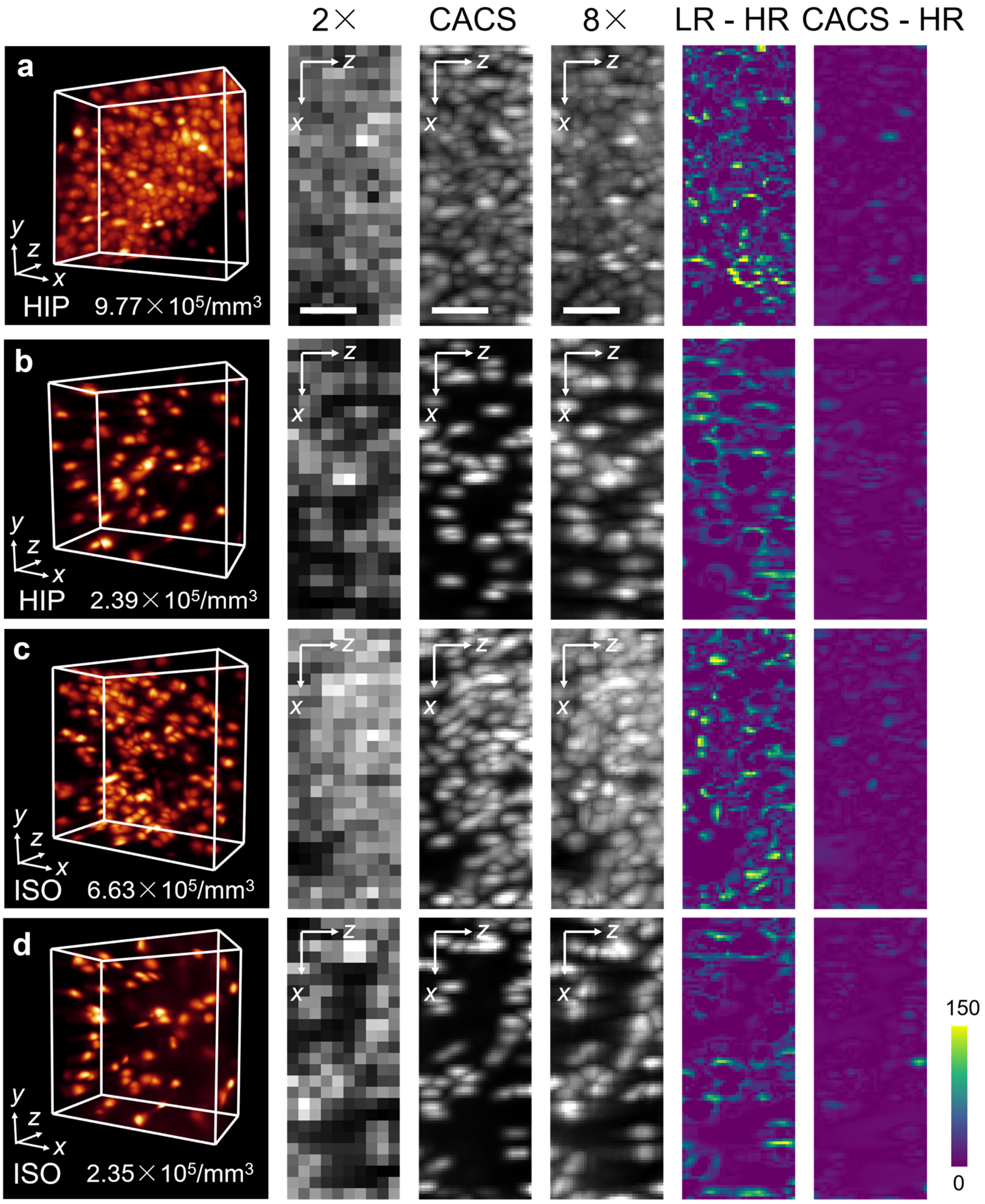

Supplementary Fig. 9 | CACS computation for point-like cell nuclei with different signal density at axial planes. a-d, Comparisons between 2×, 2× CACS and 8× results of the four volumes in PI-labelled hippocampus and isocortex, respectively. The axial planes (*x*-*z* planes) were visually inspected (2nd – 4th columns) and quantitatively evaluated (error maps in 5th and 6th columns), to validate the similarly significant improvement of axial performance in CACS results. Scale bar, 20 μm.

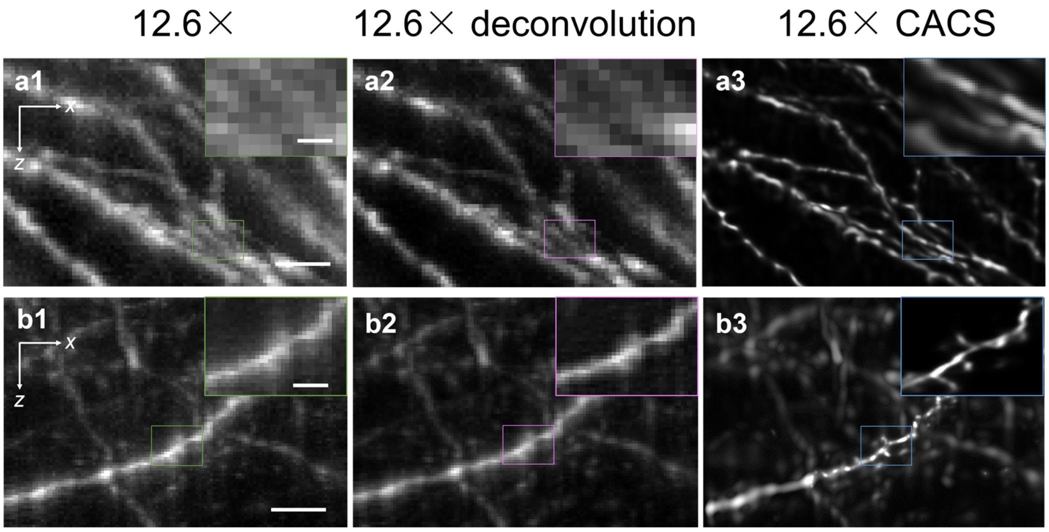

Supplementary Fig. 10 | CACS computation for resolving finer neuronal sub-structures. The CACS can be also applied to high-resolution 12.6× Bessel sheet images for super-resolving finer dendrite spines. a, b, *x*-*z* planes of two ROIs from isocortex of a Thy1-GFP-M mouse brain. As compared to the raw 12.6× Bessel sheet results (a1, b1) and deconvolution results (a2, b2), the CACS results reveals the fine structure of the dendrite spines (a3, b3). Each experiment was repeated 5 times independently with similar results. Scale bars, 10 µm (insets, 5 µm).

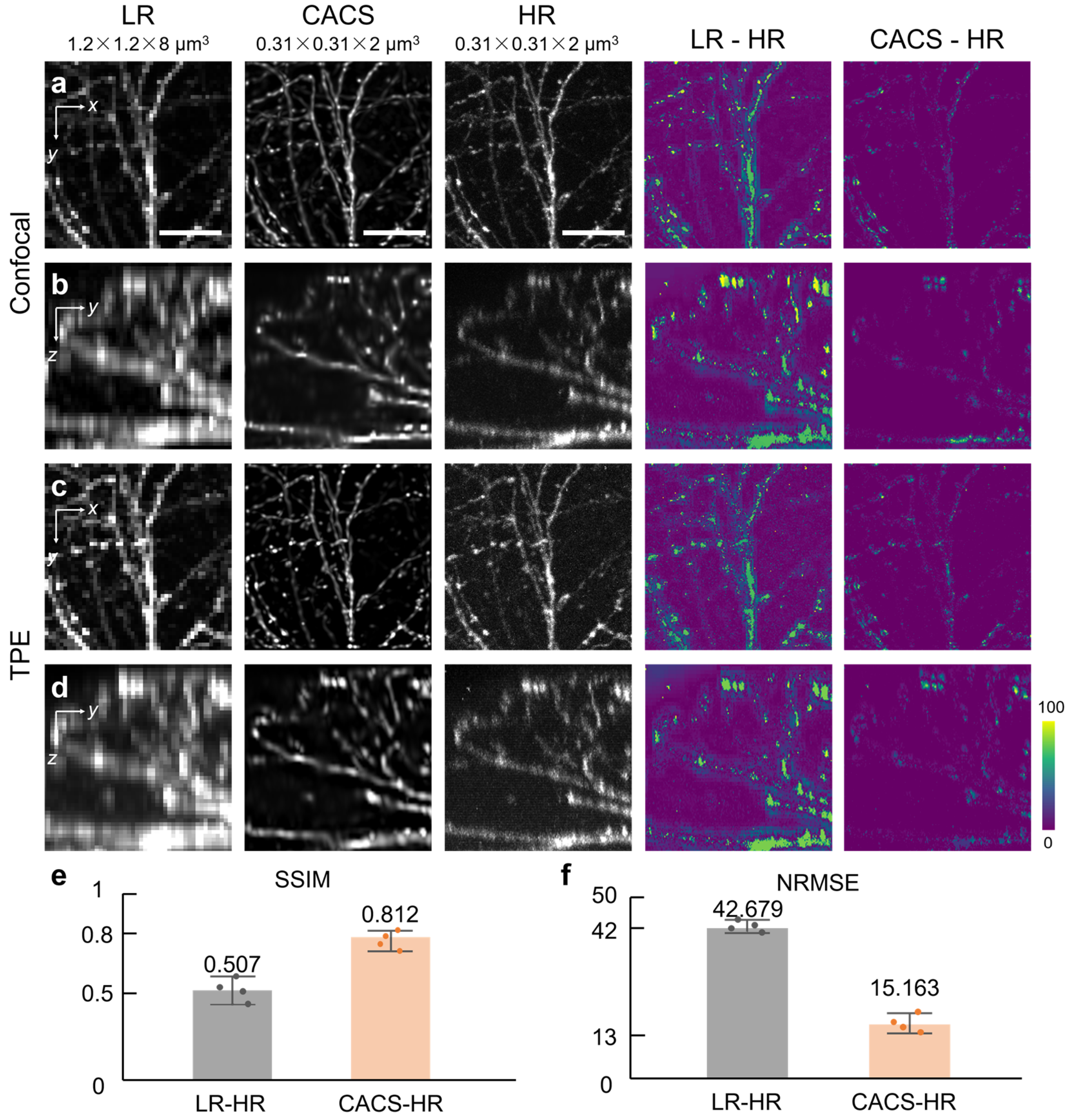

Supplementary Fig. 11 | CACS computation for images obtained by confocal and TPE microscopes. A cortex region of mouse brain (Thy1-GFP-M) was imaged using confocal and TPE microscopes. 16×/0.8W objective lens was used for collecting the fluorescence signals. The confocal and TPE images acquired by rapid coarse scanning mode and slow fine scanning mode were defined as LR (voxel size 1.2×1.2×8 μm^3) and HR (voxel size 0.31×0.31×2 μm^3) images, respectively. a-b, Comparisons between LR, CACS and HR results of the *x-y* and *x-z* planes of the confocal cortex image. c-d, Comparisons between LR, CACS and HR results of the *x-y* and *x-z* planes of the TPE cortex image. The HR results were also regarded as ground truth to validate the resolution improvement and recovery accuracy of CACS results (error maps in 4th and 5th columns). e-f, SSIM and NRMSE values of LR (with 4× bicubic interpolation) and CACS results, calculated by using HR results as reference. Data are presented as mean values +/- SD (n=4 biologically independent samples). It should be noted that when applying CACS to different microscopy modalities, we only need to change parameters related with the imaging conditions (e.g., objective NA, voxel size) to generate appropriated PSFs for initializing the computation. Scale bar, 20 μm.

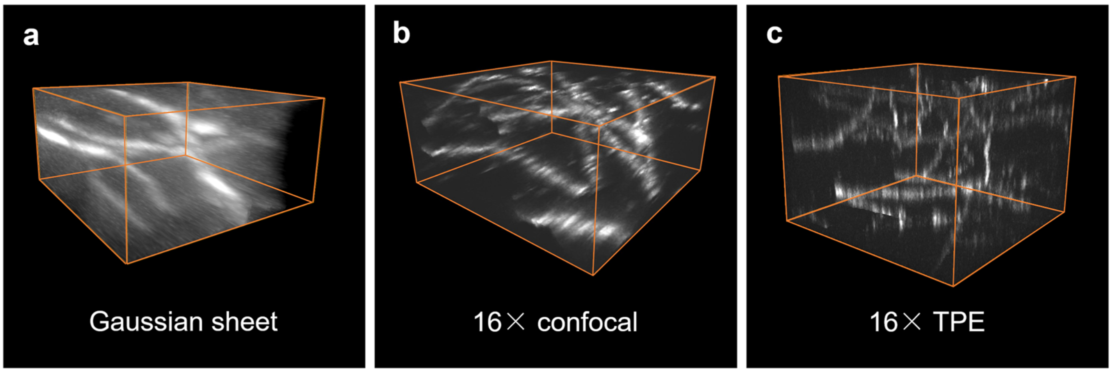

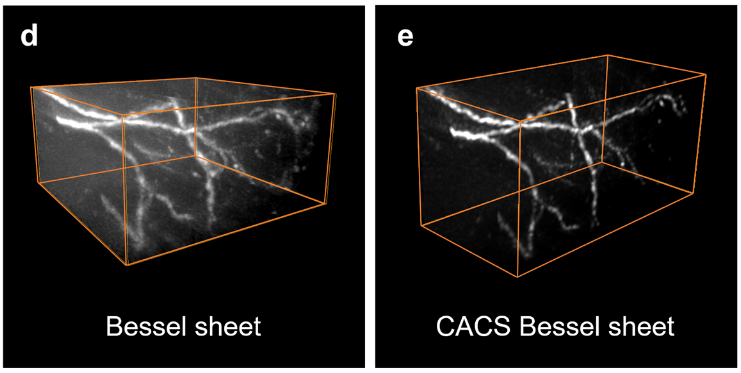

Mode	SNR
Gaussian	1.38
confocal	2.68
TPE	2.74
Bessel	2.65
CACS Bessel	20.62

Supplementary Fig. 12 | Signal-to-noise-ratio (SNR) comparison of different modes. The image SNR by each mode could be obtained via dividing the mean value of 20% brightest signal voxels in the signal stack by the root mean square (RMS) value of 20% brightest noise voxels in the noise stack (Supplementary Note 1). a-e, Image volumes of neuronal fibers in cleared brain cortex obtained by static Gaussian sheet, 16× confocal, 16× TPE, Bessel sheet, and CACS Bessel sheet, respectively. First, as compared to relatively thick optical sectioning by Gaussian sheet, the thinner and more intensive optical sectioning by Bessel sheet/confocal/TPE yields higher SNR as well as better axial resolution. Then, in addition to resolution improvement, the CS computation also notably increases the SNR of raw Bessel sheet, generating obviously highest-quality image among the five modes. All the images being compared here were obtained using regular experimental settings/parameters listed in Supplementary Note Table 1, without any denoising, such as deconvolution, applied beforehand.

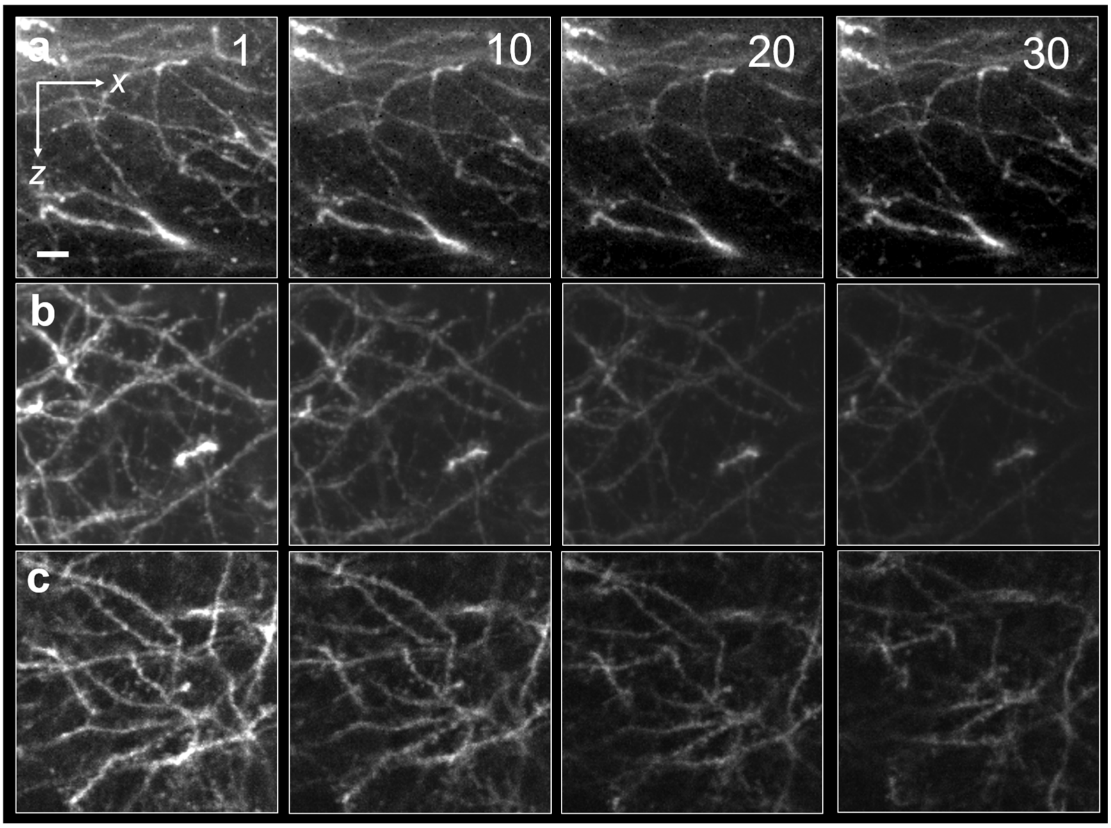

Supplementary Fig. 13 | Photo-bleaching rate comparisons of different imaging modes. a-c, Maximum-intensity-projections (MIPs) of cortex nerves in the 1st,10th, 20th and 30th image stacks by Bessel sheet, confocal and TPE, respectively. Scale bar, 5 μm. All the images were acquired by each method using parameters listed in Supplementary Table 1. Each experiment was repeated 3 times independently with similar results.

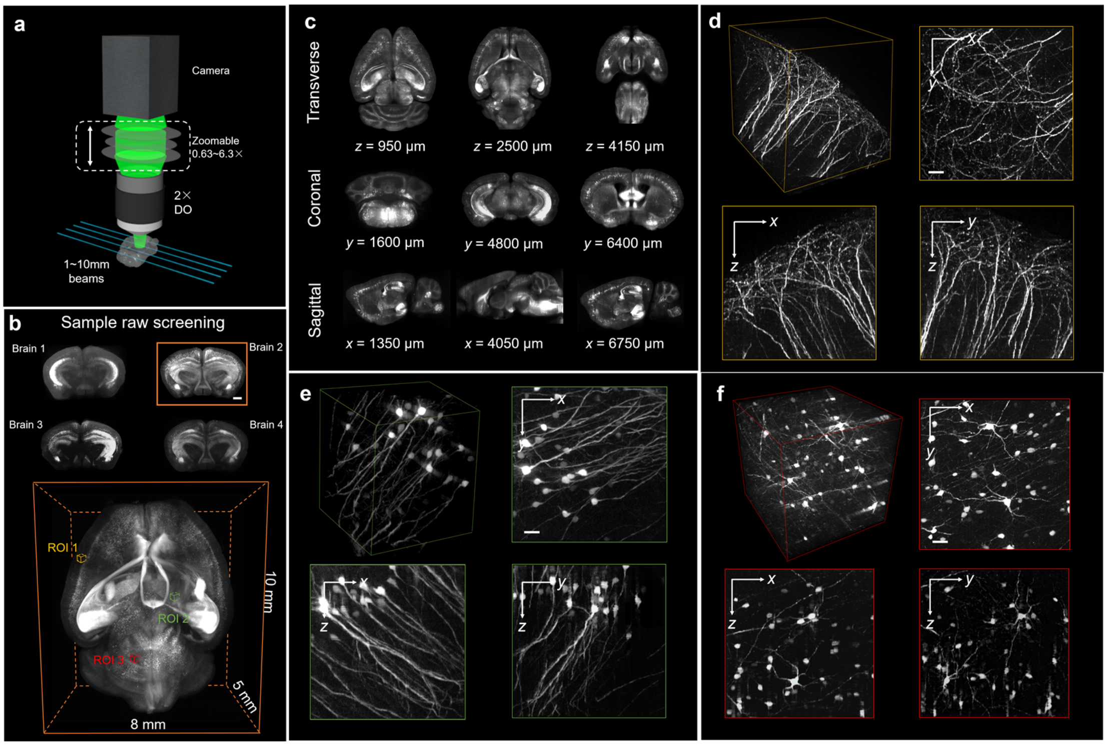

Supplementary Fig. 14 | Scalable isotropic imaging of neurons in mouse brain. a, Dual-side, tunable Bessel sheet illumination combined with zoomable detection FOV from 1.26× (~1 cm) to 12.6× (~1 mm). b, Reconstructed coronal planes of four mouse brains that were rapidly screened using raw 1.26× Bessel sheet mode. Each brain was imaged under 2 views for generating a multi-view-fused 3D reconstruction that contains 1000 image planes. The bottom row shows volume rendering of a whole brain (No.2) selected, owing to its best signal distribution among 4 candidates. c, MIPs in transverse (*x*-*y*; top), coronal (*x*-*z*; middle), and sagittal (*y*-*z*; bottom) planes of the No.2 whole brain, showing the overall signal distributions. Then, higher-resolution imaging of any region of interest (ROI) was possible using the 12.6× Bessel sheet mode. For example, we imaged three ~8×10^{-3} mm^3 regions of interest in the cortex, hippocampus, and cerebellum of No.2 brain at an imaging speed of ~0.01 mm^3 s^{-1} and an isotropic resolution of ~1.5 μm (0.5-μm voxel). d-f, Three ~8×10^{-3} mm^3 volumes in cortex (yellow), hippocampus (green), and cerebellum (red) regions, selected from the coarse 1.26× reconstruction, and further imaged by 12.6× Bessel sheet mode, to reveal the various neuron types/structures (dense dendrites in d, pyramid neurons in e, astrocytes in f) at subcellular isotropic resolution. Scale bar, 20 μm.

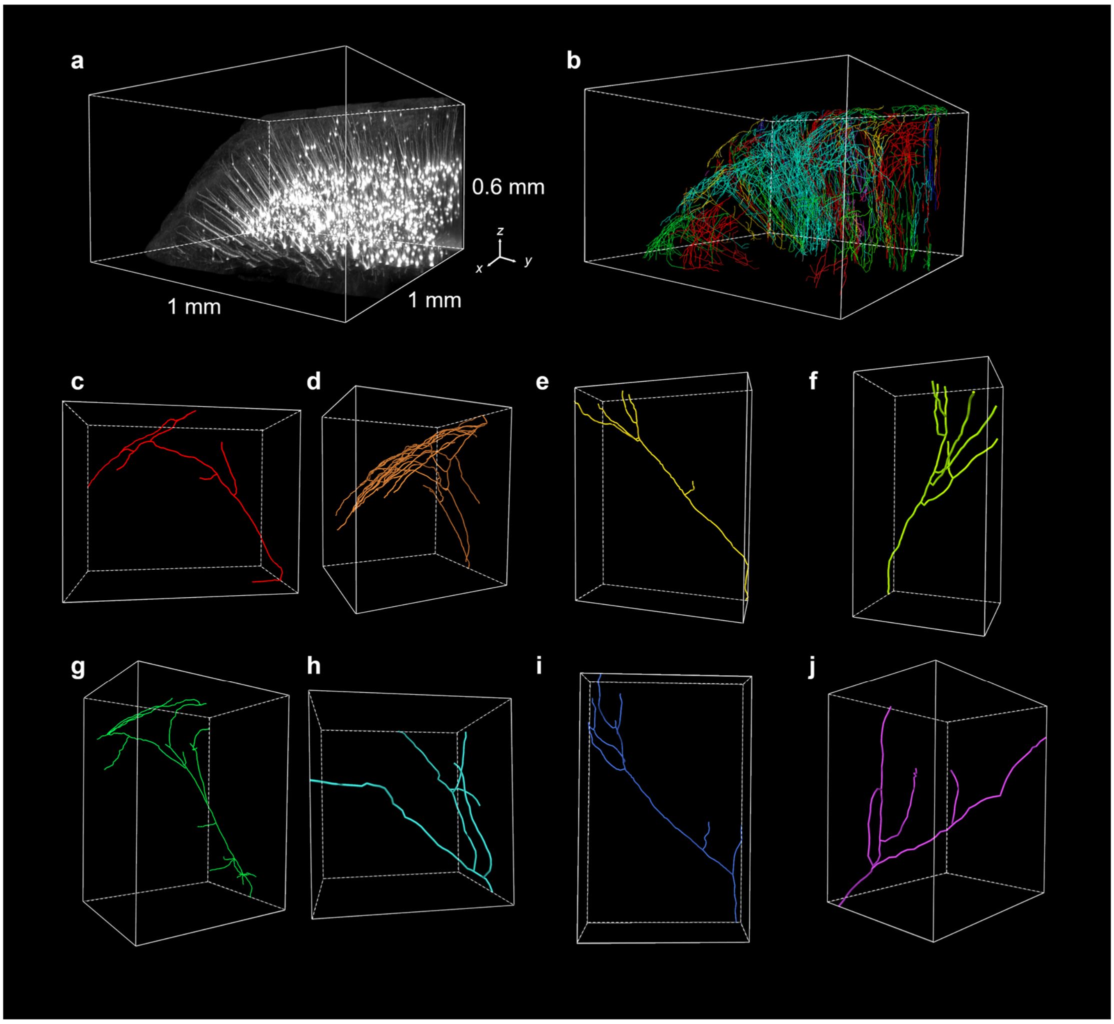

Supplementary Fig. 15 | Tracing dense neurons in the cortex area of mouse brain (Thy1-GFP-M, 8 weeks). a, 3D CACS Bessel sheet image of a cortex area ($1 \times 1 \times 0.6$ mm^3 volume) containing densely-packed pyramidal tract neurons. b, A neuronal tree with more than 200 neurons traced by applying the Neuro GPS-Tree[1] to our CACS Bessel sheet image. c-j, Detailed 3D views of 8 projection neurons initiated from this dense bundle. The results have validated that CACS image with achieving high volumetric resolution across large scale allows the automatic segmentation and tracing of dense projection neurons.

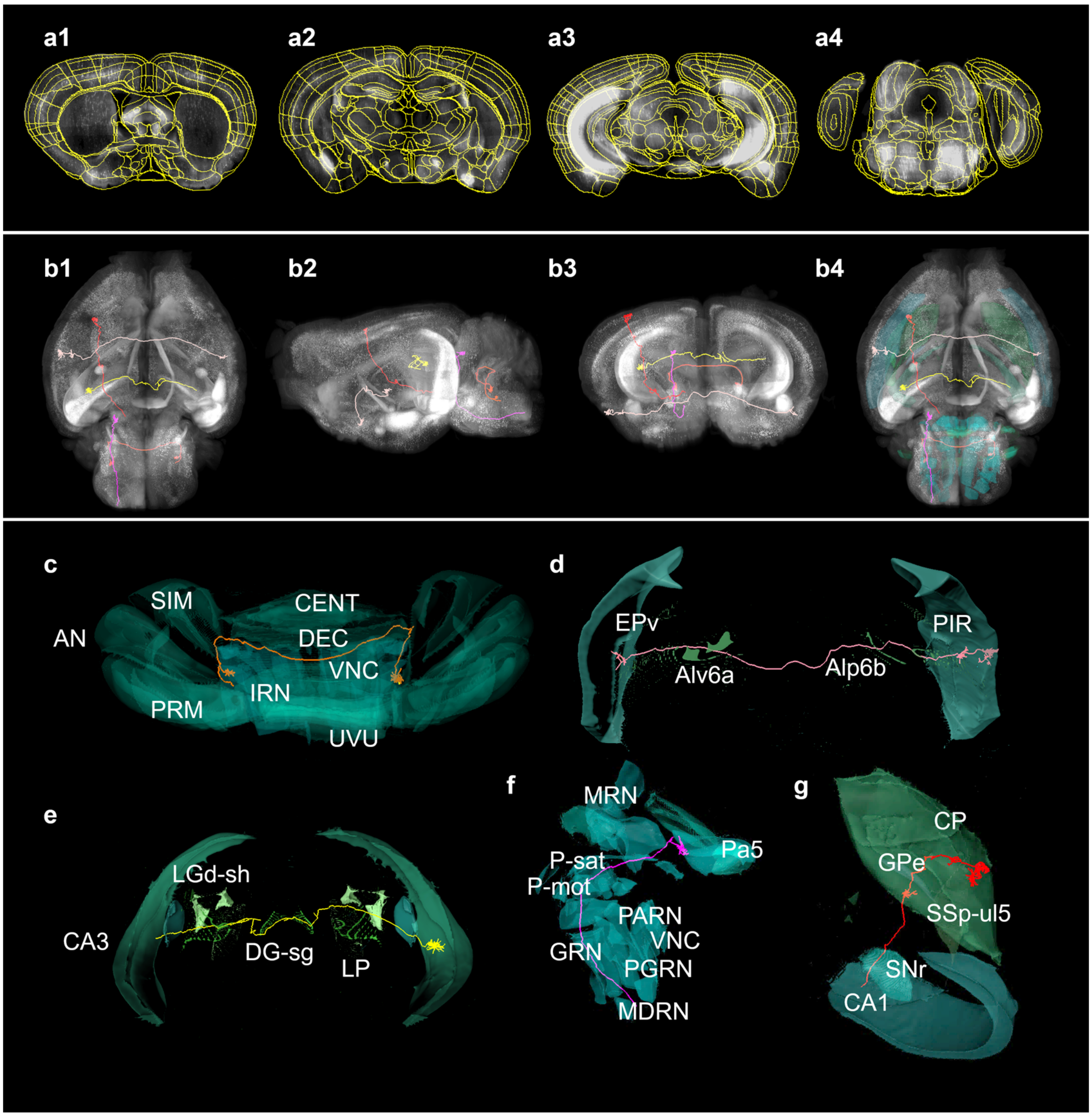

Supplementary Fig. 16 | 3D registration, region segmentation and neuron tracing of whole mouse brain (Thy1-GFP-M, 8 weeks). After imaging the whole brain using CACS Bessel sheet, we registered the whole-brain image with Allen's standard template using an accuracy-improved bi-channel image registration pipeline, which was developed by ourselves based on Elastix[2]. Then we applied the Allen partition annotation file to divide and annotate the sub-regions in the registered mouse brain. a1-a4, Registered CACS brain (shown as different coronal planes) with annotation files applied. With the creation of a digital whole-brain atlas, we could also trace the pathways of long-distance projection neurons across the entire brain. b1-b4, 3D visualization of whole brain with showing the trajectories of long-distance projection neurons (b1-b3) and the brain sub-regions they pass through (b4). c-g, Five annotated projection neurons with pathway across different regions. Ansiform lobule (AN), Simple lobule (SIM), Paramedian lobule (PRM), Intermediate reticular nucleus (IRN), Central lobule (CENT), Declive (DEC), Vestibular nuclei (VNC), Uvula (UVU) for c; Ventral part (EPv), Agranular insular area, ventral part (AIv6a), Agranular insular area, posterior part (AIp6b), Piriform area (PIR) for d; Ammon's horn (CA3), lateral geniculate complex, dorsal part (LGd-sh), Dentate gyrus, granule cell layer (DG-sg), lateral posterior nucleus of the thalamus (LP) for e; Midbrain reticular nucleus (MRN), Pons, behavior state related (P-sat), Pons, motor related (P-mot), Parvicellular reticular nucleus (PARN), Vestibular

nuclei (VNC), Gigantocellular reticular nucleus (GRN), Paragigantocellular reticular nucleus (PGRN), Medullary reticular nucleus (MDRN), Posterior amygdalar nucleus (Pa5) for f; Caudoputamen (CP), External segment (GPe), Ammon's horn (CA1), Primary somatosensory area, upper limb (SSp-ul5), Substantia nigra, reticular part (SNr) for g.

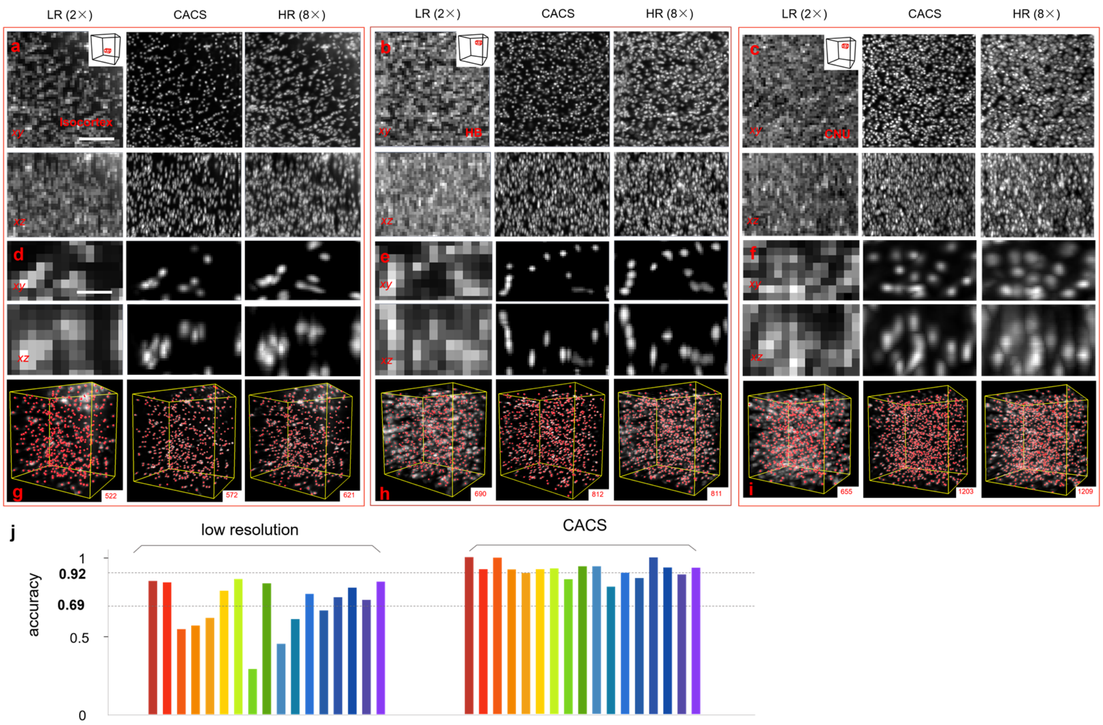

Supplementary Fig. 17 | Accuracy of compressed sensing in PI-labelled brain. We verified the high accuracy of CACS-enabled cell counting in seventeen 3D volumes from all the 10 sub-regions of a half brain. a-f, PI-labelled nuclei images (MIPs) of three selected volumes ($1 \times 1 \times 1$ mm^3) from isocortex, HB, and CNU, respectively (a-c). The signal density in these three sub-regions was highly different, thereby being suited for testing the robustness of CACS recovery. Each region was visualized by 2× Bessel sheet, 2× CACS Bessel sheet and 8× Bessel sheet, as shown in left, middle and right column, respectively. The magnified details ($32 \times 60 \times 32$ μm^3) from these sub-regions by three modes were compared to highlight the notable resolution improvement by CACS recovery (comparing d1-f1 and d2-f2), which was also highly relevant with subsequent quantitative analyses. Meanwhile, the recovered signals by 2× CACS were also verified to be sufficiently accurate (comparing d2-f2 and d3-f3). g-i, Cell nuclei segmentation in 2×, 2× CACS, and 8× images using Imaris software. j, Cell counting accuracy, defined as (2× number / 8× number) × 100% or (CACS number / 8× number) × 100%, in 2× Bessel sheet (left) and 2× Bessel-CACS (right) results of all seventeen 3D volumes. All the LR 2× images were up-sampled (4× bicubic interpolation) for comparison. Using counting results of 8× images as reference, the averaged counting accuracy in 2× results of these volumes is merely 0.69, while this value is notably increased to ~0.92, in 2× CACS results. The high accuracy of the Imaris automatic counting was verified by our visual inspection. Scale bar, 50 μm.

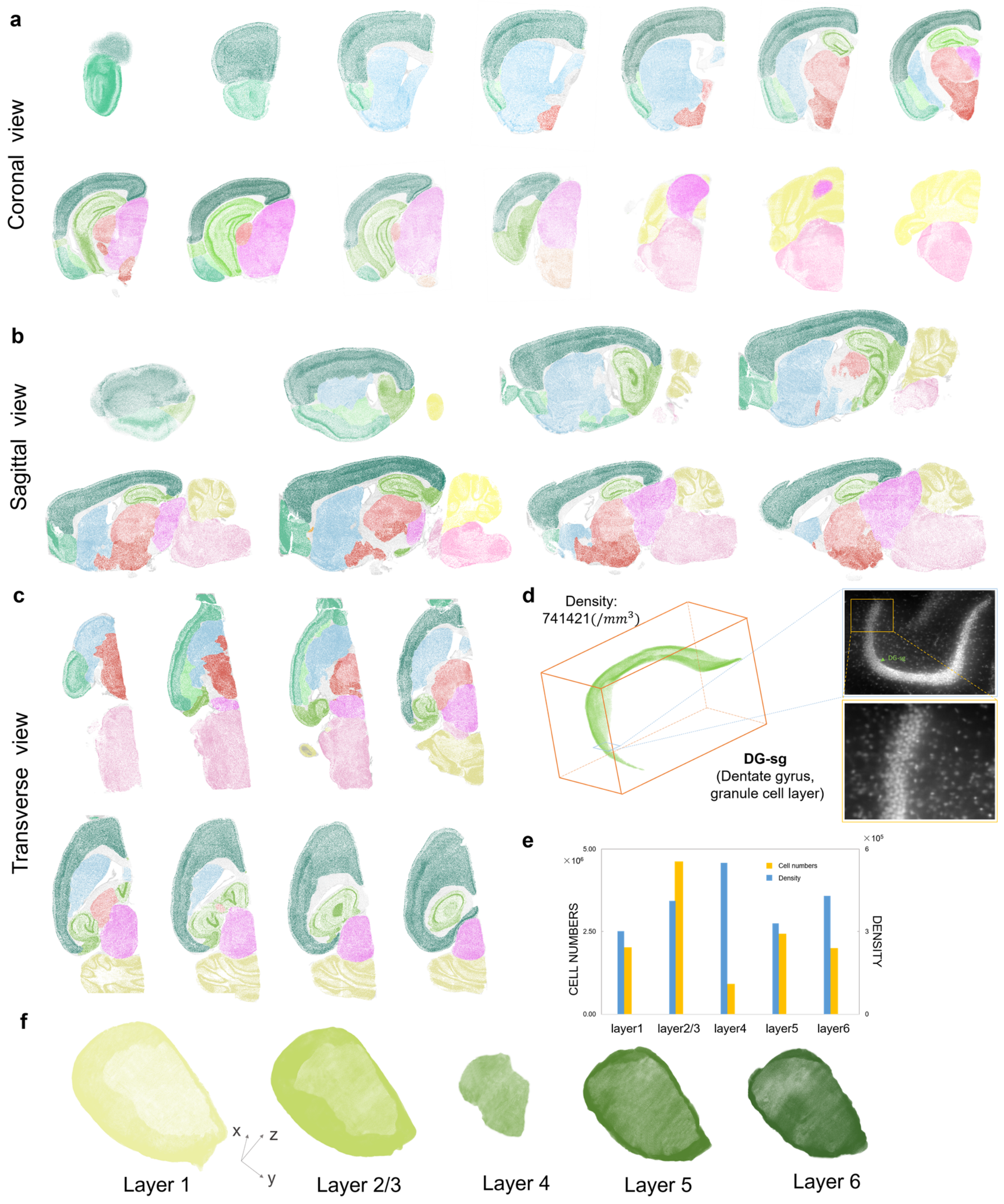

Supplementary Fig. 18 | Segmentation and cell counting for PI-labelled half brain imaged by 2× CACS Bessel sheet. With verifying the counting accuracy of CACS Bessel sheet, we applied it to the quantitative analysis of half brain. a-c, Series of coronal views, sagittal views and transverse views in different y, x and z positions, showing the segmented encephalic regions in a PI-labelled half brain. d, Selected 3D reconstruction and image details of granule cell layer at Dentate gyrus. e, f, Cell numbers and density of different layers in isocortex.

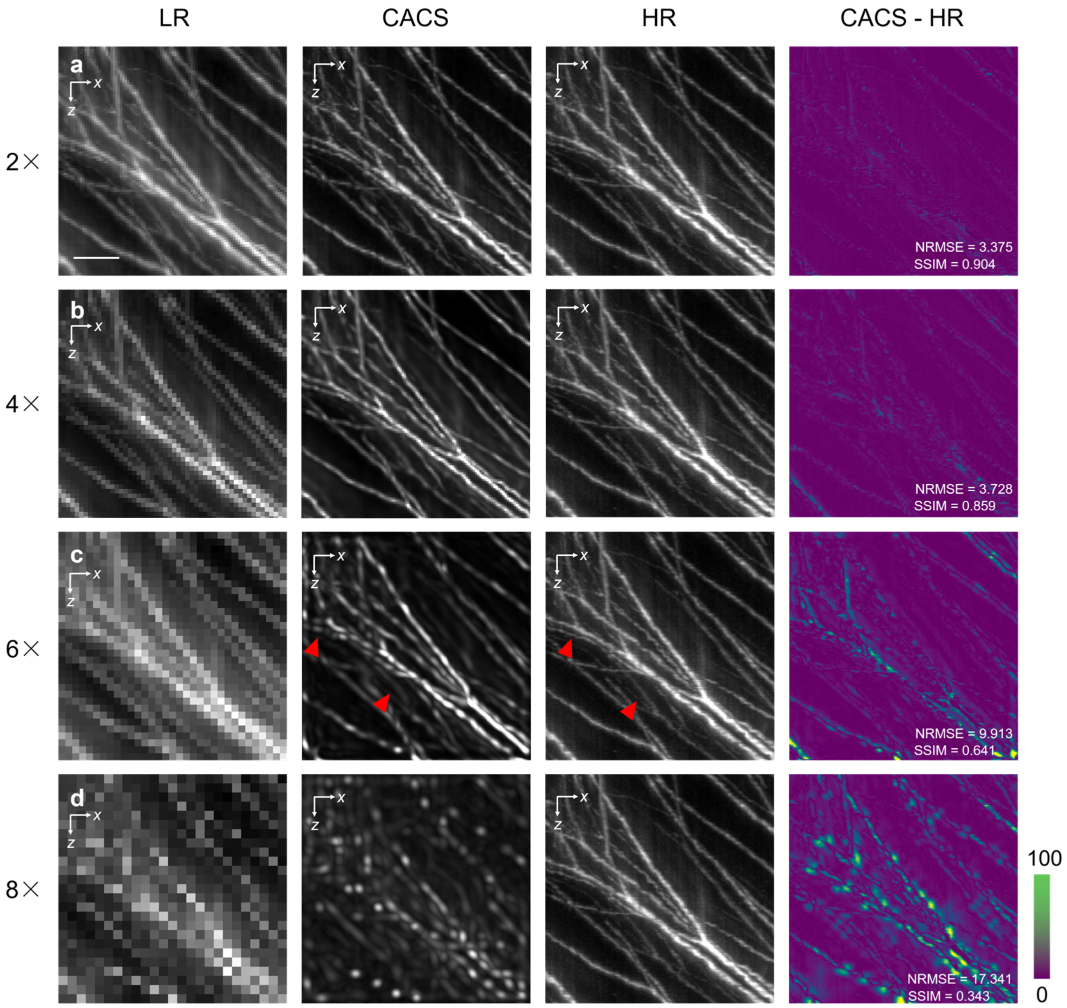

Supplementary Fig. 19 | The limit of CACS recovery. a-d, Performance of CACS when recovering the 3D neuron signals (at cortex region of Thy1-GFP mouse brain) that were down-sampled 2, 4, 6 and 8 times from the HR reference image, respectively. As shown in a-b, both 2× and 4× CACS successfully provided high-quality recovery results when compared to the HR reference images. While 6× CACS in c also showed an acceptable recovery quality in the sparse areas, noticeable artefacts arose in the dense areas (red arrows) at the same time. 8× CACS results in d failed to recover most of signals, owing to the too poor quality of LR image. The error maps between CACS and HR results shown at the right column quantitatively compared the recovery accuracy under different down-sampling rates. Each experiment was repeated 4 times independently with similar results. Scale bar, 20 µm.

Supplementary Note 1 | Imaging speed, photobleaching rate and SNR

The performances compared between the mentioned methods are listed in following table. All the comparison is based on the imaging results of mouse brain.

The speed for each method is calculated in following form:

$$\text{Imaging speed} = \frac{\text{Imaging volume}}{\text{Acquisition time}} \ (\text{mm}^3/\text{second}) \quad (1)$$

We applied a mask to remove all frequency components beyond the Abbe limit in Fourier domain to divide the raw 3D stack to a filtered stack I_1 and a noise stack I_2. Then the SNR is calculated by

$$\text{SNR} = \frac{\text{mean } (\text{Top}_{0.2} I_1)}{\text{RMS } (\text{Top}_{0.2} I_2)} \quad (2)$$

The bleaching rate curve displays the relationship between imaging times and normalized signal intensity *S*. A selected 100 μm × 100 μm × 100 μm cortex volume was repetitively imaged 30 times to compare the photobleaching rates. The signal bleaching over the time for each method is shown in Supplementary Fig. 13.

The imaging throughput is calculated by

$$\text{Imaging throughput} = \frac{\text{Number of voxels}}{\text{Acquisition time}} \ (\text{voxels/second}) \quad (3)$$

Supplementary Note Table 1 | Comparison of different imaging modes

Modes		TPE	Confocal	3.2× Gauss	3.2× Bessel	3.2× CS	12.6× Bessel
Imaging parameters	Magnification/NA	16×/0.8 (Water)		3.2×/0.28 (Air)			12.6×/0.5 (Air)
	Frame rate (fps)	1		40			20
	Pixel size (μm)	0.31 × 0.31		2.03 × 2.03		(0.508 × 0.508)	0.516 × 0.516
	Z step (μm)	2		4	2	(0.5)	0.5
Acquisition time	Speed (×10^6 μm^3/s)	0.034	0.034	2768.89	1384.37	1384.37	11.17
	Throughput (×10^6 voxels/s)	0.177		157.28		10737.42	83.88
	Photobleaching	60%	70%	5%	10%		20%
3D resolution	Lateral	0.85	0.85	4.5		1.5	1.5
	Axial	4	4.2	15	4.5	1.5	1.5

Supplementary Note 2 | Image stitching and dual-view image fusion

Cleared mouse brain (PEGASOS), or other large organs, still show non-negligible light attenuation and scattering at the deep of tissue. For dual-view 3.2× Bessel imaging of whole mouse brain, we only acquired information at 0-3 mm depth of the brain (totally ~5 mm in depth) for each view, discarding degraded signals from the deepest tissues and thereby reducing the imaging time by ~40%. 6 lateral tiles (4.16 × 4.16 × 3 mm FOV, ~40 gigavoxels) were stitched under each view, to form dual-view whole brain data. Then the complete whole-brain information could be obtained by a bead-based registration of 2 views followed by a weighted image fusion (Supplementary Note Table 2). The registered-and-fused Bessel brain was further processed by CACS to obtain the final digital whole brain with large volume size and high spatial resolution. The implementation details of the whole-brain imaging are listed below (Supplementary Note Table 2). It is noteworthy that 3.2× CACS Bessel sheet can provide subcellular resolution similar with that by 12.6× Bessel sheet while significantly reduce the acquisition time down to ~10 minutes, over 100-folds shorter than the time for 12.6× Bessel sheet.

Supplementary Note Table 2 | Whole-brain imaging with different magnification

<table>
<tr><th>Modes</th><th>1.26× Gauss</th><th>1.26× Bessel</th><th>3.2× Bessel</th><th>3.2× CACS Bessel</th><th>12.6× Bessel</th></tr>
<tr><td>Stitching tiles</td><td colspan="2">1</td><td colspan="2">2 × 3</td><td>8 × 12</td></tr>
<tr><td>Pixel size (μm)</td><td colspan="2">5.16</td><td>2.03</td><td>0.508</td><td>0.516</td></tr>
<tr><td>Z step (μm)</td><td>4</td><td>2.5</td><td>2</td><td>0.5</td><td>0.5</td></tr>
<tr><td>Frame rate</td><td colspan="4">40</td><td>20</td></tr>
<tr><td>Acquisition speed</td><td>40 s/ whole brain</td><td>60 s/ whole brain</td><td colspan="2">10 min/ whole brain</td><td>16.5 h/ whole brain</td></tr>
<tr><td>Data storage</td><td>9.76 Gb</td><td>15.625 Gb</td><td>103.9 Gb</td><td>6.49 Tb</td><td>6.07 Tb</td></tr>
</table>

Supplementary Note 3 | Content-aware regularization in CS

In CACS, an important step is to calculate the content-aware parameter λ_i which determines the weighting of the regularization term in the following equation (1), thereby affecting the solution to the signal x to be recovered

$$\min(\|Ax_i - y_i\|^2 + \lambda_i \|x_i\|_1) \quad (1)$$

The equivalent condition for Equation (1) to have a non-zero solution is $\lambda_i \in (0, \|2A^T y_i\|)$. Thus, we can define $\lambda_i = \|2A^T y_i\| \cdot \beta_i$, with $\beta_i \in (0, 1)$. As we further make $\|2A^T y_i\| = \alpha_i$, the regularization term can be written as $\lambda_i = \alpha_i \cdot \beta_i$. As we have shown in the data plot in Supplementary Note Fig. 1c, the value of α_i was found to have negative linear correlation with the signal density in y_i (signal voxel number / total voxel number). Therefore, it can be considered as a signal density indicator with larger α_i indicating sparser signals in y_i. However, the signal density alone remains inadequate to completely characterize the signal distribution, also not enough to determine the λ_i. Thereby, we also introduced the degree of signal disorder to distinguish the cases, in which the averaged signal density might be similar but the signal uniformity (distribution) is actually very different. A common representation of the degree of signal disorder is the entropy. We first selected a number of y_i to calculate their entropy E using the *entropy* function in Matlab[3]. In these test data, the entropy for point-like nuclei signals varied from 3 to 4.8, while this value was smaller from 0 to 4 for line-like neuron signals which can be regarded as the orderly arrangement of point signals. Then we empirically tried different β_i for these y_i, and identified the optimal one for each y_i according to the best recovery results. Then the obtained E and β_i for these selected y_i were linearly correlated via a curve fitting expressed as $\beta_i = k \cdot E_i + b$, where k = -0.08 and b = 0.52 for point-like signals, such as cell nuclei, and k = -0.35 and b= 1.82 for line-like signals, such as neuronal fibers (Supplementary Note Fig. 1d). Then the parameter β_i for all the input y_i can be automatically determined from the curve, with larger β_i denoting less-disordered signals in y_i. Thus, here β_i is considered as a negative indicator for the degree of signal disorder. Finally, an appropriate weighting factor $\lambda_i = \alpha_i \cdot \beta_i$ for each y_i determined according to the signal characteristics, and allows adaptive CACS reconstruction of optimal x_i from various types of y_i (Supplementary Note Fig. 1a, b). Such a content-aware regularization is especially necessary for large-scale image recovery. In conventional CS implementation, the recovery of dense signals tends to have over-fitting issue caused by excessive initial constraints, thus showing poor improvement. On the contrary, the recovery of sparse signals require more constraints to prevent too sharp artefacts[4]. Because the signals across a large FOV varies dramatically, conventional CS implementation with fixed regularization unsurprisingly causes obvious artefacts and signal-loss in the final stitched result (Supplementary Note Fig. 1e). The content-aware regularization factor λ_i introduced in our CACS procedure can properly judge the signal characteristics and dynamically balance the results between these two extremes, thereby recovering relatively accurate signals with substantial resolution improvement (Supplementary Note Fig. 1f, g). For example, as the calculated λ_i being large, also meaning sparse and ordered signals presented in raw image, the algorithm correspondingly has large weighting from the raw image and tends to preserve more existing real information rather than inference of new information.

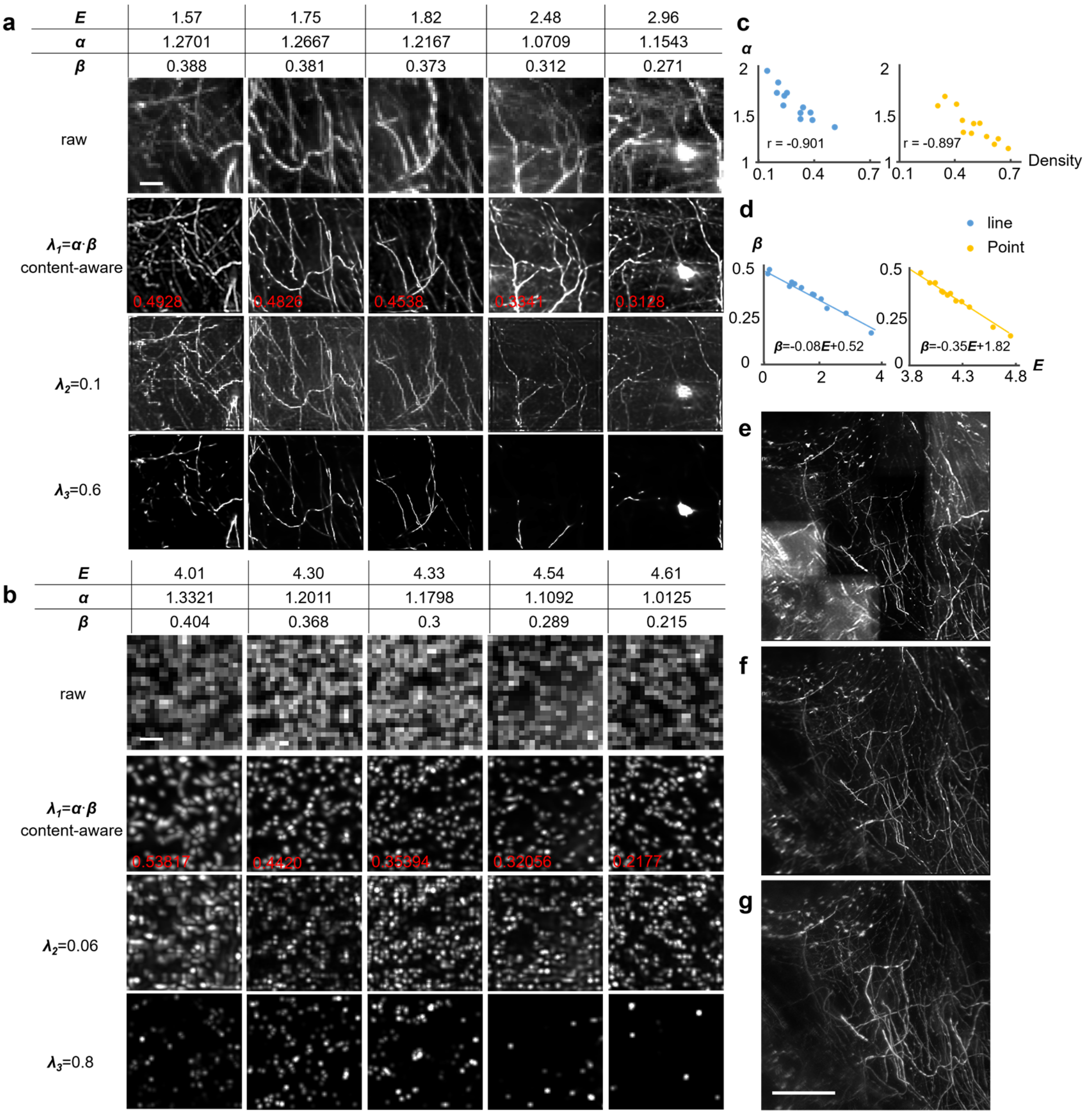

Supplementary Note Fig. 1 | Content-aware calculation of regularization factor. a, 5 line-like structures from Thy1-GFP-M mouse brain with applying different sparsity indicator α and entropy indicator β. Three types of CS enhancement results with $\lambda_1 = \alpha \cdot \beta$, $\lambda_2 = 0.1$ and $\lambda_3 = 0.6$ are compared to show the artefacts for λ_2, information loss for λ_3, and obviously higher accuracy by CACS. b, 5 point-like structures selected from PI-labelled mouse brain also verify the advantage of CACS. Scale bar, 20 µm. c, The data plot of parameter α versus the signal density, indicating the negative linear correlation with the signal density in y_i. d, The plot of parameter β versus the entropy of signals (E). The fitted curves (solid lines) reveal the inversely proportional relationship between β and the entropy, in both point- and line-like signals. e-g, Comparison of conventional CS computation with constant parameters (e), our CACS with adaptive parameters (f), and 12.6× ground-truth image (g). Scale bar, 100 µm.

Supplementary Note 4 | Synthetic PSF and measurement matrix A in CACS

First a PSF image in spatial domain was generated based on the transfer function of our Bessel imaging system. Then the measurement matrix A, also the Fourier form of PSF, was obtained to correlate the low-resolution measurement with high-resolution image to be recovered in Fourier space. According to NA of detection objective in our Bessel microscope, we used a 3D Gaussian function with appropriate kernel size to represent the optical blurring of a point source by system (Step 1 in Supplementary Note Fig. 2). The axial intensity distribution of the blurred point was then modulated using a Bessel function with parameters matching the property of real Bessel beam, to simulate the Bessel plane illumination with side-lobe effect included (Step 2). We removed the high-order side lobes according to the width of the camera's rolling shutter, to simulate the side-lobe-rejection effect by electronic confocal slit (Step 3). This tailored PSF kernel was down-sampled (pixel average) into a 3D PSF image with voxel size matching the acquisition setup, e.g., $2 \times 2 \times 2$ μm^3 voxel corresponding to 3.2× camera detection + 2-μm z-scan step size (Step 4). Finally, the measurement matrix A was generated through a Fourier transformation of the synthetic PSF image (Step 5).

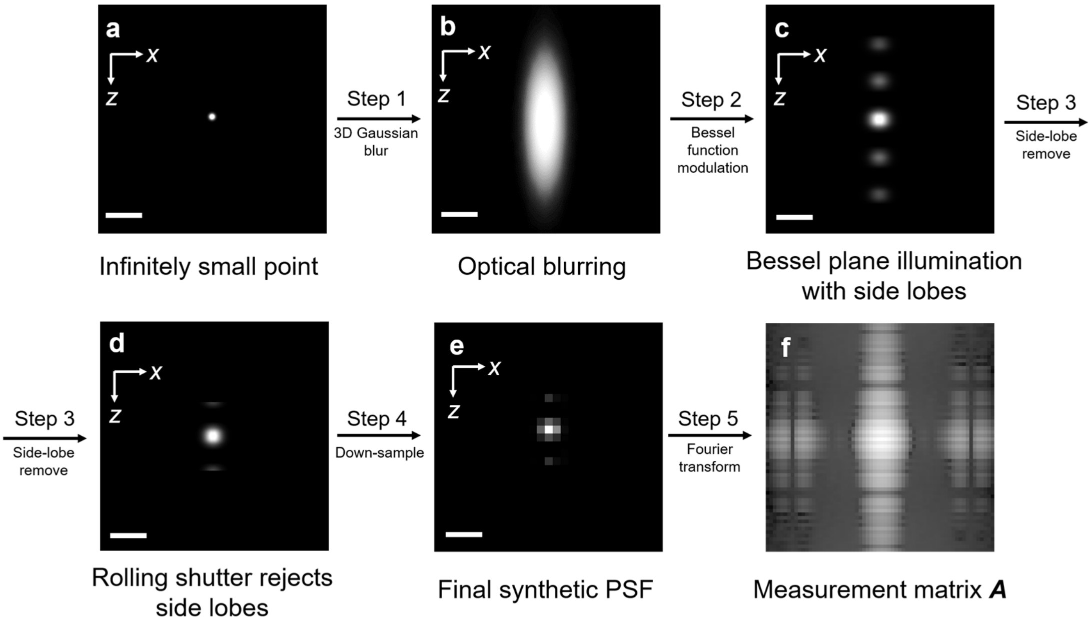

Supplementary Note Fig. 2 | Generation of synthetic PSF and measurement matrix A through modeling the Bessel-sheet imaging process. a-d, Five steps that model the Bessel-sheet imaging process are applied sequentially to generate the system PSF and the measurement matrix A. The obtained A is then used in CACS to correlate the low-resolution measurement with high-resolution image to be recovered in Fourier space. Scale bar, 5 μm.

Supplementary Note 5 | Iteratively solving the minimization problem using interior-point method

A well-established interior-point method[5] was used to iteratively solve the equation (1) in the following form:

$$x_{i,n+1} - x_{i,n} = \|Ax_i - y_i\|_2^2 + \lambda_i \|x_i\|_1 \quad (2)$$

To calculate the specific iteration step and the convergence threshold, the general idea is to transform Equation (1) to its dual equation G(v_i) = $\frac{1}{4}((v_i^T v_i) - v_i^T y_i)$, where $v_i = 2s_i\,(Ax_i - y_i)$ is the dual argument of x_i, s_i, as the iteration step, is calculated by $s_i = min\{\lambda_i/(2A^T(Ax - y))_i\}$. We also calculate the duality gap η, which is the difference between Equation (1) and G(v_i). The duality gap would decrease as the iteration progresses. As the duality gap becomes small enough (difference between two equations approaching 0), Equation (1) and G($\boldsymbol{v_i}$) form a strong dual relationship and the solving process reaches its convergence. After a large number of image tests, we set the convergence threshold $\boldsymbol{\varepsilon}$ to be 0.01, with which the computation time and improvement effect are well balanced. For detailed principles and mathematical derivation, please refer to the reference 5.

The specific steps for CACS are given below:
Input parameters: voxel sizes, interpolation factor and raw image Y_i;
Initialization parameters: relative tolerance $\varepsilon = 10^{-2}$; lateral and axial sigma of Gaussian blur; z-crop size; $X_{i,0}$ (the bicubic interpolation of Y_i) as the initial image for the first iteration;

Program running:
Step 1: Calculate α_i, β_i and λ_i by the raw image Y_i;
Step 2: Generate a synthetic PSF by voxel sizes, interpolation factor, Gaussian blur and axial crop, then apply a Fourier transformation of PSF to obtain A;
Step 3: Repeat the iteration of the interior-point method for solving the Equation (2):
Repeat:
3.1 Compute the search direction by preconditioned conjugate gradient (PCG) method;
3.2 Compute the iteration step s_i by backtracking line search method;
3.3 Update $x_{i,n+1} = x_{i,n} + s_i$;
3.4 Construct dual feasible point v_i and evaluate duality gap η;
3.5 Quit if $\eta/G(v_i) \le \varepsilon$;
3.6 Obtain the final x_i.
Step 4: Inverse Fourier transform of x_i into the spatial domain to obtain the CACS result X_i.

References in Supplementary Information

1. Quan, T. et al. NeuroGPS: automated localization of neurons for brain circuits using L1 minimization model. *Scientific Reports* **3**, 1414 (2013).
2. Wang, X. et al. Bi-channel Image Registration and Deep-learning Segmentation (BIRDS) for efficient, versatile 3D mapping of mouse brain. *bioRxiv*, 2020.2006.2030.181255 (2020).
3. Thum, C. Measurement of the Entropy of an Image with Application to Image Focusing. *Optica Acta: International Journal of Optics* **31**, 203-211 (1984).
4. Cherkassky, V. & Mulier, F. Learning from data : concepts, theory, and methods. (2007).
5. Koh, K., Kim, S.-J. & Boyd, S. An interior-point method for large-scale L1-regularized logistic regression. *Journal of Machine Learning Research* **8**, 1519-1555 (2007).
