# Supplementary material for: Minutes-timescale 3D isotropic imaging of entire organs at subcellular resolution by content-aware compressed-sensing light-sheet microscopy
Source: Nat Commun. 2021 Jan 4;12:107. doi: 10.1038/s41467-020-20329-3 (PMC7782498; doi:10.1038/s41467-020-20329-3)
Supplement: Supplementary file 2 — Description of Additional Supplementary Files [file 41467_2020_20329_MOESM2_ESM.pdf]

Title: Supplementary Movie 1

Description: Imaging process of Bessel light-sheet microscopy. The confocally-scanned mode was compared to global shutter mode to demonstrate the effect of side-lobe rejection. A 3D volume from Thy1-GFP-M mouse brain was imaged by global shutter mode and confocally-scanned mode, respectively. The achieved axial resolutions were compared in *y*-*z* planes.

Title: Supplementary Movie 2

Description: Content aware compressed sensing (CACS) procedure, including the compressed matrix *A* and the specific iterative optimization steps. A comparison was made between the conventional CS and CACS when computing a large low-resolution 3D volume of neuron signals, showing that CACS dynamically balance the results between over-fitting and under-fitting by adjusting the regularization factor.

Title: Supplementary Movie 3

Description: Axial coronal slices of the Thy1-GFP-M transgenic whole mouse brain by 3.2× Bessel sheet and 3.2× CACS Bessel sheet. The 3D visualization from two sub-volumes demonstrated the resolution improvement by CACS.

Title: Supplementary Movie 4

Description: Sequential transverse planes and 3D visualization of the Thy1-GFP-M whole mouse brain. The magnified views of cellular structures from different sub-regions (CB, Isocortex, CNU, HB, HPF and MB) were shown.

Title: Supplementary Movie 5

Description: 3D segmentation of a whole mouse brain based on the high-quality image by CACS Bessel sheet microscopy.

Title: Supplementary Movie 6

Description: Demonstration of neuronal tracing in mouse brain. 13 neurons from the cortex sub-region were accurately segmented and traced.

Title: Supplementary Movie 7

Description: 5 long-distance neuronal projections across the entire brain were traced by Imaris semi-automatically. The sub-regions these neurons passed through were annotated with different colors.

Title: Supplementary Movie 8

Description: CACS improved the 3D resolution of point-like cell nuclei from PI-labelled mouse brain. 3D visualization and cell counting results from two sub-volumes validated the resolution improvement by CACS.

Title: Supplementary Movie 9

Description: Sequential transverse planes and 3D visualization of the PI-labelled mouse brain. Cellular structures from sub-regions (Isocortex, CNU, HB, HPF and MB) were shown.

Title: Supplementary Movie 10

Description: Segmentation of the PI-labelled mouse brain. The cell counting showed the number and density of nuclei in each specific sub-region.

Title: Supplementary Movie 11

Description: 3D visualization of two gastrocnemius and two tibialis muscles from mouse. The motor endplates (MEP) were labelled by α-BTX and the peripheral nerves were labelled by Thy1-YFP. Four muscles were imaged by dual-color CACS Bessel sheet microscopy (488 for peripheral nerves and 637 for MEPs).

Title: Supplementary Movie 12

Description: 3D imaging, visualization and quantitative analysis of a gastrocnemius. The Neuron tracing and MEP counting based on the dual-color images followed the same strategy applied in mouse brain analysis. The comparison between LR and CACS results of three MEPs indicated the resolution improvement by CACS.
